# Decoding chronic pain: the glutamate-GABA tug of war in the cerebral cortex

**DOI:** 10.3389/fnmol.2025.1572775

**Published:** 2025-07-23

**Authors:** Dan Huang, Yu-Ting Dong, Liu-Xuan He, Rui-Zhu Zhou, Jian-Xiong Zhou, Sha Yang, Shu-Guang Yu

**Affiliations:** ^1^School of Acupuncture and Tuina, Chengdu University of Traditional Chinese Medicine, Chengdu, China; ^2^Key Laboratory of Acupuncture for Senile Disease, Chengdu University of TCM, Ministry of Education, Chengdu, China; ^3^Acupuncture and Chronobiology Key Laboratory of Sichuan Province, Chengdu, China

**Keywords:** chronic pain, cerebral cortex, glutamatergic system, GABAergic system, neural network

## Abstract

A sustained imbalance between excitatory and inhibitory mechanisms within the glutamatergic and GABAergic systems of the cerebral cortex, induced by noxious stimuli, is a fundamental characteristic in the development and maintenance of chronic pain. This review provides a comprehensive summary of the roles and interaction of glutamatergic and GABAergic systems in the processing of chronic pain signals. Specifically, we present a systematic summary of the processing patterns of the cerebral cortex in the cross-modular integration and output of chronic pain information, according to four aspects, molecular, cellular, neural network and behavioral cognition. These patterns consist of neuronal responses in individual cortical regions, neuron-astrocyte interactions, sharing and cascading of inter-cortical signals, and downward cortical modulation. Furthermore, a number of potential therapeutic approaches to the chronic pain are discussed from the pain management perspective.

## 1 Introduction

The cerebral cortex, the highest level of central nervous system (CNS), governs sensory-motor functions and emotional responses. Its neural networks excitability is regulated by two key systems: the glutamatergic system and the gamma-aminobutyric acid (GABA) system (Mazo et al., 2022; Wu et al., 2023; Ganguly et al., 2001; Kami et al., 2022; Petroff, 2002; Bell et al., 2021). The glutamatergic system directly encode sensory information and reshapes cortical nerve fiber projections (Tan et al., 2020; Waqas et al., 2018; Cui et al., 2022), whereas the GABAergic system modulates excitatory inputs and optimizes the representation and processing of supra-modal information (Ferguson and Gao, 2018; Tremblay et al., 2016). While the GABAergic system modulates excitatory input and refines the processing of external stimuli, thereby maintaining accurate sensory perception (Tan et al., 2020). The dynamic fluctuations of these systems can, to some extent, reflect the current excitability state of the brain.

Continuous exposure to noxious stimuli can lead to a dysregulation of the balance between excitatory glutamatergic and inhibitory GABAergic systems in the cerebral cortex, which is progressively exacerbated. This impairs normal cortical excitability and sensory output of the noxious sensory network, resulting in neurological network instability, aberrant information transmission (Chen et al., 2022; Marcello et al., 2013; Eto et al., 2012; Chang et al., 2014; Benson et al., 2015) and abnormal nociception (Pigott et al., 2023). Meanwhile, cortical circuits undergo functional modifications and a reduction in their capacity for corrective action (Cheriyan and Sheets, 2020), which ultimately results in an enhanced processing of chronic pain (Pigott et al., 2023; Watson, 2016; Mecca et al., 2021; Zhang et al., 2015; Peek et al., 2020; Niddam et al., 2021; Kang et al., 2021). This process also evoke negative emotions, which interactively exacerbate chronic pain (Song et al., 2024; Li et al., 2023). A cerebral excitation-inhibition (E/I) imbalance is considered a significant factor in the development of pain (Eto et al., 2012; Watson, 2016; Cao et al., 2019), as well as a typical feature of the transition from acute to chronic pain (Bell et al., 2021; Pigott et al., 2023; Peek et al., 2020; Wang et al., 2022; Zunhammer et al., 2016) and the long-term maintenance of chronic pain (Zunhammer et al., 2016; Thiaucourt et al., 2018). This imbalance is also a critical factor in the development and maintenance of central sensitization of the brain (Watson, 2016; Raja et al., 2020; Lyu et al., 2021; Bhatt et al., 2023; Li et al., 2019). Furthermore, there is a positive correlation between the ratio of glutamate to GABA and pain sensitization (Zunhammer et al., 2016; Thiaucourt et al., 2018).

The extant review literature focuses on individual systems, drawing systematic conclusions about for dysregulation of the glutamatergic system (Temmermand et al., 2022; Zanetti et al., 2021; Yang and Chang, 2019; Ozawa et al., 1998) or the GABAergic system (Li et al., 2019; Qian et al., 2023; Fischer, 2017; Comitato and Bardoni, 2021) in chronic pain. Furthermore, it addresses distinctive pain treatment modalities that target the systems and mechanisms that may be associated with the onset or maintenance of chronic pain. Moreover, some literatures have reported the progressive functional and structural changes in the brain that occur in individuals with chronic pain (Yang and Chang, 2019) and changes involve different neuronal types and complex neural networks that process pain information (Kuner and Kuner, 2021; Tan and Kuner, 2021; Mercer Lindsay et al., 2021). However, there is a lack of systematic knowledge regarding temporal dynamics in the E/I balance and the dynamic processing of pain information in the cerebral cortex at the level of the glutamatergic and GABAergic dual systems.

Therefore, this review considers the glutamatergic and GABAergic systems, which are vital for sustaining the typical E/I equilibrium of the brain, as a foundational focus. It exhibits a detailed analysis of the chronological changes in interaction between glutamatergic and GABAergic systems in pain and the processing patterns of pain information for the cerebral cortex, involving various dimensions of chronic pain. The objective is to achieve a systematic understanding of the role of the cerebral cortex in the cross-modular integration and output of chronic pain information at four levels, namely molecular, cellular, neural network and behavioral cognition. Moreover, therapeutic strategies targeting various elements of the glutamatergic and GABAergic systems are classified to facilitate new perspectives into pain treatment.

## 2 Cortical processing of pain-related information

Pain is a multidimensional sensory experience involving sensory, cognitive, and affective components (Raja et al., 2020). Research focusing on the spinal cord, as the site for receiving, integrating, and gatekeeping primary pain signals, is insufficient to fully elucidate the mechanisms underlying the development and persistence of chronic pain, or to account for its cognitive and affective components (Comitato and Bardoni, 2021; Rankin et al., 2024; Kiani et al., 2024; Zheng et al., 2020). The cortex integrates incoming nociceptive stimuli, extracts key pain features and re-encodes this information (Ziegler et al., 2023; Ishikawa et al., 2023; Guo et al., 2023; Xiao et al., 2023; Gan et al., 2022; Li et al., 2022; Hu et al., 2019; Shao et al., 2023). It then mobilizes hierarchically organized neuronal projections to diverse brain regions (Li et al., 2023; Guo et al., 2023; Li et al., 2022; Chen et al., 2024; Hu et al., 2021; Smith et al., 2021; Cardoso-Cruz et al., 2019; Garcia-Larrea and Peyron, 2013), engaging in bidirectional interactions with glial cells (Kim et al., 2016; Zhou et al., 2025). This dynamic process establishes a cortex-centric pain modulation network. This network governs the propagation and feedback of pain-related information across the brain, orchestrates responses to the multifaceted components of pain, and ultimately drives adaptive changes in behavior, cognition, and emotional state (Gan et al., 2022; Li et al., 2022; Martins et al., 2015; Falconi-Sobrinho et al., 2017; Singh et al., 2020; Buetfering et al., 2022).

Furthermore, sustained nociceptive input progressively worsens the E/I imbalance between glutamatergic and GABAergic systems within the cerebral cortex, leading to functional reorganization (Sanganahalli et al., 2021; Baliki et al., 2012; Obermann et al., 2013). This reorganization is characterized by a spatiotemporal shift in brain activity, transitioning from sensory structures toward anterior emotional and limbic regions (Baliki et al., 2012; Park et al., 2022; Li et al., 2025). Substantial evidence indicates this renders neural networks more susceptible to persistent activation across all pain dimensions (Sanganahalli et al., 2021). Consequently, this elevated and prolonged network activity underlies the prolongation of pain duration, maintenance of the pain state, and the development of associated emotional and cognitive impairments and motor dysfunction (Weizman et al., 2018), thereby promoting pain catastrophizing (Gnall et al., 2025; Korkut, 2025; Jee et al., 2023). Critically, the degree of E/I imbalance and its associated shift in the cortex may reflect the brain’s integrated representation of the perceived intensity of the ongoing chronic pain state, concurrent emotional alterations, prognostic predictions, and the processing mechanisms underlying chronic pain itself (Baliki et al., 2012).

## 3 Brain regions exhibiting time-varying E/I balance and divergent neural response

**Figure 1:**
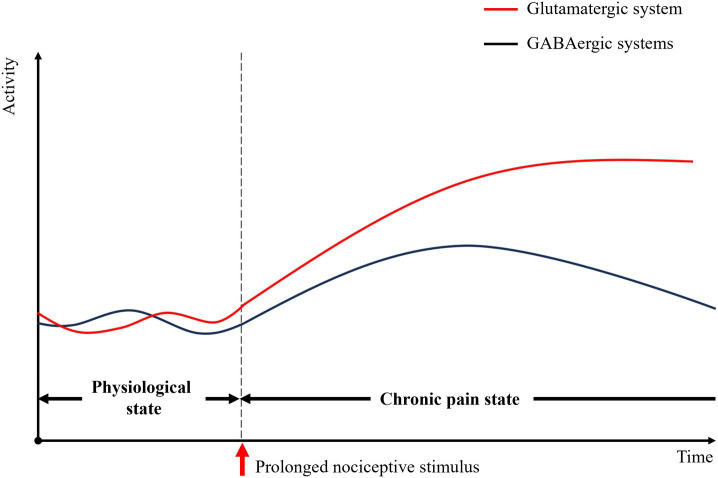
The dynamic changes of glutamatergic and GABAergic system. In physiological state, the two systems work together in response to external stimuli, in part as a function of the current state of arousal. In chronic pain state, prolonged exposure to noxious stimuli can induce sustained excitation of the glutamatergic system in the cerebral cortex. The excitability of the GABAergic system changes from increased to decreased to compensate for the abnormal change in excitability of the CNS. However, this is not sufficient to restore normal homeostasis.

Persistent nociceptive input directly targets cortical regions responsible for pain perception and emotional processing. This induces a sustained E/I imbalance, triggering dysregulated of neuronal gene expression, aberrant receptor signaling, and disruption of downstream transduction pathways. Consequently, maladaptive synaptic plasticity develops, which disrupts normal sensory encoding and leads to pathological processing of nociceptive signals. These alterations result in impaired discrimination of noxious stimuli and distorted pain perception, while simultaneously generating negative affective states (as illustrated in Figure 1).

The glutamatergic system plays a dominant role on the initial processing of pain. Noxious stimuli enhance glutamatergic transmission (Cseh et al., 2020), leading to a reflexive increase in glutamate levels (Watson, 2016; Wu et al., 2008; Guida et al., 2015). Concurrently, changes occur in the structure and function of glutamate receptors, such as N-methyl-D-aspartate receptors (NMDARs) (Mao et al., 2022; Zugaib et al., 2014; Fan et al., 2018; Sun et al., 2016; Hogrefe et al., 2022; Ren et al., 2022; López-Avila et al., 2004), *α*-amino-3-hydroxy-5-methyl-4-isoxazolepropionic acid receptors (AMPARs) (Sun et al., 2016; Ren et al., 2022; Chen et al., 2014; Liu et al., 2015) and metabotropic glutamate receptors (mGluRs) (Cao et al., 2019; Kim et al., 2016; Ji and Neugebauer, 2011; Notartomaso et al., 2024), inducing synaptic plasticity. This plasticity manifests as alterations in the induction of long-term potentiation (LTP) (Tullis et al., 2023) or the suppression of long-term depression (LTD) (Liu W. et al., 2024). Collectively, these changes result in functional hyperactivity of cortical glutamatergic neurons and widespread neuronal excitation. Evidence shows that following nerve ligation or complete Freund’s adjuvant (CFA) injection, glutamatergic neurons in the secondary somatosensory cortex (S2) (Guo et al., 2023), anterior cingulate cortex (ACC) (Zhang et al., 2017), insular cortex (IC) (Zhang et al., 2023), and prelimbic cortex (PL) (Ma L. et al., 2024) exhibit hyperactivity. This hyperactivity contributes to an imbalance in E/I dysfunction, an increased sensitivity to pain (Wu et al., 2008; Chen et al., 2014; Tullis et al., 2023) and an enhanced aversion behavior (Zhang et al., 2017). Furthermore, excessive excitation of glutamatergic neurons in these pain-processing regions can inhibit glutamatergic neurons in other brain areas (Thompson and Neugebauer, 2019; Ji et al., 2010), thereby prioritizing the perception and transmission of nociceptive signals within the brain.

The modulation of GABAergic system dynamically shifts during chronic pain progression, evolving from an adaptive inhibitory state to a maladaptive disruption of e E/I balance. The GABAergic system acts to counteract neuronal excitation. Co-activated alongside the glutamatergic system, GABAergic activity serves as a marker for persistent changes in pain perception. Painful stimuli have been observed to stimulate GABA release (Jasmin et al., 2003; Peek et al., 2021), enhance GABA_A_ receptor activation via mGluR1 (Ji and Neugebauer, 2011) and promote GluD1 binding—an iGluR family member—with GABA (Piot et al., 2023). Consequently, GABAergic neurons are activated, partially correcting the E/I imbalance (Eto et al., 2012; Zhang et al., 2023). However, the effect of enhanced inhibition is proves insufficient to fully counterbalance the increased glutamatergic excitation and alleviate chronic pain symptoms (Eto et al., 2012; Cao et al., 2019; Zhang et al., 2023). Paradoxically, pain itself can directly hyperactivate the GABAergic system, triggering feedforward inhibition that reduces output neuronal activity, thereby promoting pain persistence (Zhang et al., 2015; Kim et al., 2024). Conversely, when pain becomes sustained or intensifies, the GABAergic system becomes dysregulated. This leads to a further breakdown of E/I balance, facilitating the development of persistent pain sensitization and the emergence of diverse complex pain-related behavioral comorbidities.

Furthermore, a disrupted collapse of the GABAergic system occurs in the absence of the removal of exogenous or endogenous stimuli, resulting in a malignant E/I imbalance, persistent pain sensitization and the emergence of a variety of complex pain-related behaviors. It has identified that inhibitory synaptic deficits in hippocampus (HPC) induces by the increase of α5-containing GABA_A_ receptors (Cai et al., 2022) or GABA release (Saffarpour et al., 2017) mediate cognitive impairment associated with pain. A reduction of the inhibitory synaptic transmission resulting from the decrease in GABA release and expression of vesicular GABA transporter, although the GABA transporter-1 increasing in neuropathic pain (Masocha, 2015), can contribute to the development of pain and anxiety in the ACC in inflammatory pain (Shao et al., 2021; Koga et al., 2018). Interestingly, the addition of exogenous GABA can reverse abnormal excitability caused by paclitaxel-induced neuropathic pain in the ACC (Nashawi et al., 2016). Conversely, the over-release of GABA, induced by the loss function of CB1R in GABAergic neurons of the PL, can lead to depression (Mecca et al., 2021; Li et al., 2022).

Critically, pain responses exhibit significant heterogeneity even among neurons within a single brain region, underscoring the complexity of cortical pain processing. Distinct subregions and diverse subtypes of glutamatergic and GABAergic neurons within a given area respond differentially to pain signals. In the medial prefrontal cortex (mPFC) of mice, neuropathic injury increases the excitability of parvalbumin-expressing neurons in layer 5 of the PL subdivision (Jones and Sheets, 2020). Conversely, it reduces the excitability of somatostatin-expressing neurons in layers 2/3 (Jones and Sheets, 2020). Painful stimuli attenuate the excitability of glutamatergic neurons in the anterior but not in inferior limbic subregions of the mPFC (Cheriyan and Sheets, 2018). In the midcingulate cortex (MCC), the majority of glutamatergic neurons in zone 2 are inhibited by painful stimuli. Whereas zone 1, they are activated (Hu et al., 2019). Cortical responses to distinct types of pain stimuli also exhibit region-specific heterogeneity. Glutamatergic neurons in the ventral hippocampal CA1 (vCA1) subregion exhibit increased excitability in response to neuropathic pain (Kami et al., 2022), but conversely show reduced excitability following CFA-induced inflammatory pain (Shao et al., 2023). Concurrently, glutamatergic neurons in the dorsal hippocampus also demonstrate decreased excitability during inflammatory pain (Wei et al., 2021). Moreover, distinct subregions of a single brain region can mediate the information transmission of different components. The anterior parts of the IC preferentially mediate somatosensory features of pain, while the posterior parts are more involved in the affective features (Meng et al., 2022). Glutamatergic neurons in layers 5 and 6 of the primary motor cortex (M1) encode downstream projection information for the various components of pain (Gan et al., 2022).

## 4 Functional homeostatic of neuron-astrocyte interaction

It is clear that the neuron-astrocyte crosstalk is critical for maintaining the normal function of the nervous system. Astrocytes play a critical role in the regulation of synaptic gap homeostasis, which is instrumental in the perception and maintenance of central sensitivity to chronic pain (Ozawa et al., 1998; Kim et al., 2016; Lee et al., 2022). This is achieved by forming the tripartite synapse with neurons (Araque et al., 1999; Perea et al., 2009), involving in the glutamate-glutamine cycle, generating and regulating the release of glutamate (de Ceglia et al., 2023) and GABA (Koh et al., 2023; Vélez-Fort et al., 2012; Cheng et al., 2023) and their own activity in response to stimuli (Cahill et al., 2024). This interaction is disrupted in chronic pain, resulting in an over-excitation of neurons and a diminished inhibitory effect of astrocytes on neurons. This further exacerbates the imbalance between the excitation and inhibition in the brain, leading to disturbances in pain signaling and processing and excitatory neurotoxicity (Ozawa et al., 1998; Lee et al., 2022).

Astrocytes can influence neuronal activity through signaling mechanisms. Sustained nociceptive stimulation induces aberrant glutamate metabolism, which activates astrocytes to drive structural and functional alterations in neuronal synapses via tripartite synaptic connections. These changes provoke localized hyperexcitability within the somatosensory cortex and trigger circuit rewiring, ultimately disrupting pain-processing cortical networks and culminating in chronic pain pathogenesis (Kim et al., 2016; Kim et al., 2017; Danjo et al., 2022). Thus, cortical astrocyte activation both modulates nociceptive input and promotes synaptic plasticity changes, such as dendritic spine formation in the cortex. This process disrupts the maladaptive neural connections formed in the cortex during the transition from acute to chronic pain (Takeda et al., 2022). Notably, a similar phenomenon is observed in the ACC. Sustained noxious stimulation induces hyperactivation of astrocytes. This astrocytic hyperactivation promotes pain persistence and the emergence of anxiety-like behaviors by increasing synapse-related protein expression (Cardoso-Cruz et al., 2019), re-expressed mGluR5 (Shen et al., 2025) or upregulating the signal pathway for S100B (the astrocyte marker S100 calcium binding protein B)-RAGE (The receptor for advanced glycation end-products) (Jiang et al., 2025), thereby altering excitatory neuronal plasticity and enhancing neuronal activity via tripartite synapses. Intriguingly, astrocytes not only directly modulate E/I balance in local brain region (Wei N. et al., 2024), but also influence downstream pain-processing brain regions via neuronal circuit from the ventrolateral orbitofrontal cortex (vlOFC) to the ventrolateral periaqueductal gray (vlPAG) (Islam et al., 2025), thereby contributing to pain persistence.

In addition, astrocytes couple neural activity to energy metabolism. In the ACC, reduced glutamatergic metabolism and impaired glutamate reuptake induce NMDA spike generation in pyramidal neuron dendrites. This stimulates astrocyte activation and proliferation, promoting pain persistence and lowering pain thresholds (Takeda et al., 2022; Romanos et al., 2020). Furthermore, astrocytes can directly modulate pain hypersensitivity (Reid et al., 2025) and pain-related aversive behaviors (Iqbal et al., 2022) through the lactate shuttle. Visceral hypersensitivity can also trigger lactate release from ACC astrocytes, contributing to decision-making deficits comorbid with chronic visceral pain (Wang et al., 2017).

## 5 Sharing and cascading of inter-cortical signals

**Figure 2:**
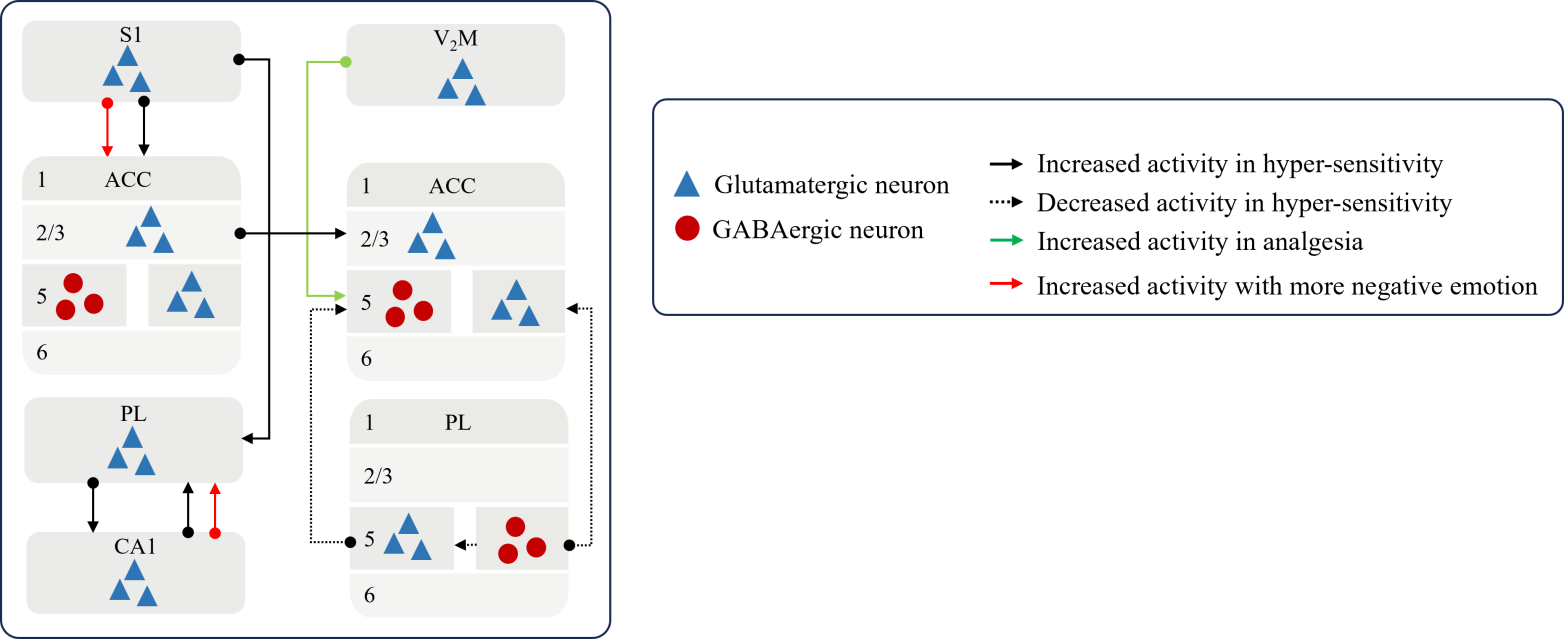
The pattern of sharing and cascading of inter-cortical signals Noxious stimulus signal is transmitted between sensory and affective centers to affect the state of painsensation and emotion. S1, the primary somatosensory cortex; V2M, the secondary visual cortex; ACC, anterior cingulate cortex; PL, prelimbic cortex; CA1.

Noxious stimulus information is shared across the cerebral cortex and transmitted between sensory and affective centers, as well as between ipsilateral and contralateral brain regions. This intercortical communication mediates synchronized activation of local cortical circuits during pain processing. Importantly, these circuits integrate feedback inhibition and facilitation, undergoing recurrent cycles of signal amplification, cascading recruitment, and dynamic rebalancing. This reverberatory process ultimately promotes the transition to chronic pain (as illustrated in Figure 2). Ipsilateral stimuli can alter the activity of structures involved in pain modulation on the contralateral hemisphere. In individuals with chronic pain, the projection from the ipsilateral ACC (Li et al., 2023; Hu et al., 2021) to the contralateral side of the brain exhibit aberrant activation, which in turn induces primary and secondary nociceptive hypersensitivity (Garcia-Larrea and Peyron, 2013). This plays an important role in the development and maintenance of chronic pain and promotes peripheral and central sensitization, which suggests that the chronic pain state of the organism is more difficult to correct. Additionally, it impairs the feedback loops for the processing of injurious information and disrupts the E/I balance between the cerebral cortex. During chronic pain, the glutamatergic neurons in PL are sustainably activated by the glutamatergic neurons in the primary somatosensory cortex (S1) (Ma L. et al., 2024), the glutamatergic neurons in the ACC are inhibited by decreasing GABAergic interneurons in PL (Li et al., 2022), or the inhibition effect of GABAergic neurons in the Zona incerta (ZI) is reduces by MCC Cg2 glutamatergic neurons (Hu et al., 2019). This leads to loss of cortical–cortical inhibition and an exacerbated and spontaneous pain.

In contrast, the restoration of a balanced excitation-inhibition equilibrium and information feedback within neural circuits between brain regions has been demonstrated to induce resistance to noxious stimuli and reinstate cortical inhibition. Activation of glutamatergic projections from the medial part of the secondary visual cortex (V2M) to GABAergic neurons in the ACC (Cao et al., 2023) and from the MCC Cg2 to GABAergic neurons in the ZI mitigate pain hypersensitivity, while transient activation of the latter also relieves aversive behaviors associated with spontaneous persistent pain (Hu et al., 2019).

Concurrently, the sensory and affective centers constantly engage in a continuous bidirectional interaction, resulting in the malignant crosstalk in pain sensation with emotion to affect individual’s coping style and emotional state. Enhanced connectivity from the primary somatosensory cortex (S1) to the anterior ACC has been observed, wherein S1 encodes sensory pain signals and the ACC processes the affective consequences of pain, thereby the aversions to nociceptive responses to enrich the negative experience of pain specificity in affective centers (Singh et al., 2020).

## 6 Downward cortical modulation

**Figure 3:**
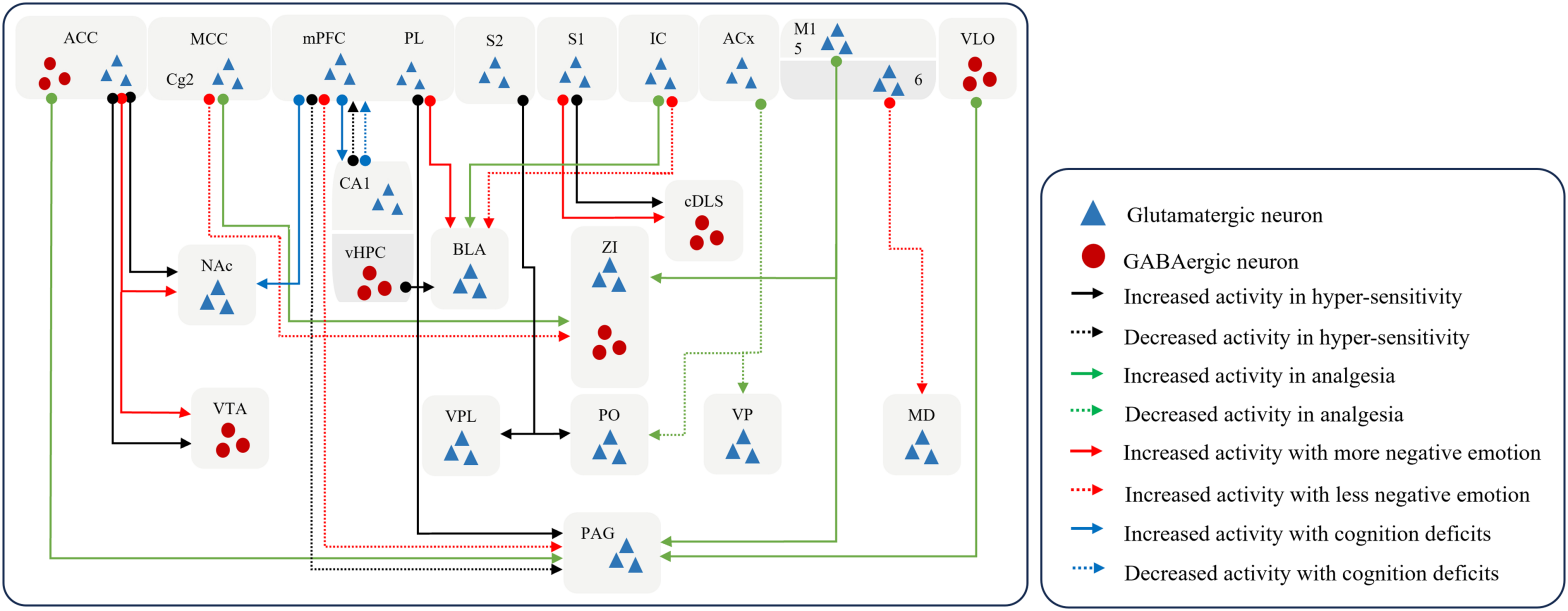
The pattern of downward cortical modulation. The cerebral cortex controls signal propagation by recognizing sensory signals and outputting perceptual signals. And prolonged injurious stimulation can remodel neural networks, leading the cortex to assign additional negative coding to stimulus signals, which in turn leads to the emergence of complex behaviors. S1, the primary somatosensory cortex; S2, the secondary somatosensory cortex; M1, the primary motor cortex; PL, prelimbic cortex; dmPFC, the dorsal medial prefrontal cortex; ACC, anterior cingulate cortex; MCC, the midcingulate cortex; IC, the insular cortex; ACx, the auditory cortex; VLO, the ventrolateral orbital cortex; vHPC, the ventral hippocampus; NAc, the nucleus accumbent; ZI, the zona incerta; VPL, the ventral posterolateral thalamic nucleus; PO, the posterior thalamic nucleus; VP, the ventral posterior nucleus; MD, the mid-dorsal thalamus; VTA, the ventral tegmental area; BLA, the basolateral amygdala; cDLS, the caudal dorsolateral striatum; PAG, the periaqueductal gray.

Higher central processing in the cerebral cortex converts noxious sensory signals into perceptual signals and input to downstream brain regions (shown in Figure 3). On the one hand, the cerebral cortex controls the flow of afferent sensory stimuli to the downward regions (Ong et al., 2019; Wu et al., 2020; Huo et al., 2005; Yin et al., 2020) by projecting sensory signals downstream to produce downstream elicitation or inhibition. An abnormally large number of perceptual signals are generated and delivered to the downstream analgesic system, whose function and homeostasis are disrupted, eventually resulting in the persistence of pain (Gan et al., 2022; Cheriyan and Sheets, 2018).

It has been shown that the disruption of connections of glutamatergic neuronal connections, from the mPFC to the periaqueductal grey (PAG) (Cheriyan and Sheets, 2018), from layer 5 in the M1 to the ZI and PAG (Gan et al., 2022), and from the S2 to the ventral posterolateral thalamic nucleus (VPL) and posterior thalamic nucleus (PO) (Guo et al., 2023) induce sensory hypersensitivity. In contrast, restoration of normal regulation of cortex over downstream brain regions can disrupt the chronic pain cycle, which can block ascending nociceptive input and induces analgesia (Yin et al., 2020; Lucas et al., 2011). The voluntary exercise can activate the GABAergic projections from the ventral HPC to the basolateral amygdala to produce the effect of hypoalgesia and alleviate negative emotions (Minami et al., 2023). Excitation of GABAergic connections from the M1 (de Andrade et al., 2019), ventrolateral orbital cortex (VLO) (Wu et al., 2020; Huo et al., 2005) to the vlPAG may facilitate the proper functioning of the downstream inhibitory system and block further upload of nociceptive information to higher centers. This processing induces analgesia at the spinal cord level. Similarly, the activation of the glutamatergic projection from the dorsal medial prefrontal cortex (dmPFC) to the vlPAG (Yin et al., 2020) and the inhibition of the inputs from the auditory cortex (ACx) to the PO and the ventral posterior nucleus (VP) (Zhou et al., 2022) and from PL (Gao et al., 2023) to vlPAG has also found to relieve nociceptive hypersensitivity.

On the other hand, the cerebral cortex induces a protective response from the organism either by directly producing pain or by generating negative emotions to facilitate the organism’s rapid departure from the environment in which the source of the nociceptive stimulus is located (Chen et al., 2024; Botvinik-Nezer et al., 2024; Kragel et al., 2018). When removed from the worse environment, the organism reverts to a state of normalcy. Hence, the production of negative emotions can be considered a predictive behavior of pain perception. Prolonged hyperexcitability of cerebral cortex results in a pain-specific negative experience. It amplifies the perception of pain and affects the pathways of pain processing in the brain, while causing abnormalities in the nociceptive modulation system and promoting the formation of pain memories (Song et al., 2024; Gan et al., 2022; Meng et al., 2022; Yin et al., 2020; Gao et al., 2023; Zeng et al., 2021).

Glutamatergic projections from the S1 to the caudal dorsolateral striatum (cDLS) (Jin et al., 2020), the M1 to the mid-dorsal thalamus (MD) (Gan et al., 2022), the dmPFC to the vlPAG (Yin et al., 2020), the IC to the basolateral amygdala (BLA) (Meng et al., 2022), the PL to the BLA (Gao et al., 2023) or to the nucleus accumbent (NAc) (Zeng et al., 2021), and the ACC to the ventral tegmental area (VTA) (Song et al., 2024) confer negative emotional messages and promote pain persistence and chronicity. Furthermore, the glutamatergic connections from the IC to the BLA is involved in the formation and consolidation of empathic pain (Zhang et al., 2022). And glutamatergic projection from the ACC to the nucleus accumbens (NAc) is intimately associated with the genesis and induction of empathic pain, in addition to the social transfer of pain and fear (Smith et al., 2021).

As established above, chronic pain involves a shift in brain activity from sensory regions to limbic regions. Pain chronicity leads to persistent activation of the limbic system. Following intense nociceptive stimulation, the limbic cortex becomes unstable and undergoes reorganization, exhibiting structural plasticity during the sustained phase of nociception (Kim et al., 2011). The brain comprehensively encodes and integrates pain perception, pain-related affect, and cognition (Talbot et al., 1991). Chronic pain imbues sensory signals with affective valence via cortico-limbic pathways, facilitating a maladaptive sensory-affective interplay that promotes long-term pain persistence. Furthermore, prolonged affective disturbances disrupt processing of cognitive information within the limbic system—including core networks for emotion and memory processing (mPFC, ACC, hippocampus, amygdala)—culminating in progressive and complex cognitive-behavioral abnormalities (Cai et al., 2022; Shackman et al., 2011). These cognitive impairments, in turn, further exacerbate emotional deterioration.

PL and the CA1 in the HPC are the primary regions for the brain associated with the formation of pain memory and cognitive process (Stegemann et al., 2023). Sciatic nerve injury can inactivate the mPFC via glutamatergic synaptic inhibition in the BLA-mPFC pathway, leading to decision-making deficits (Ji et al., 2010). Conversely, the mPFC itself can induce deficits in pain-related working memory—without affecting pain perception—through hyperactivation of the glutamatergic mPFC-dCA1 circuit (Cardoso-Cruz et al., 2019; Cardoso-Cruz et al., 2013a). Furthermore, the prelimbic mPFC can drive this dysfunction through its glutamatergic projections to the NAc (Cardoso-Cruz et al., 2022), or, in the context of chronic inflammatory pain, via glutamatergic circuitry projecting to the mediodorsal thalamus (Cardoso-Cruz et al., 2013b). Similarly, chronic pain induced by sciatic nerve injury also can impair spatial learning and memory by increasing GABA release and decreasing glutamate levels in the HPC (Saffarpour et al., 2017). Furthermore, it mediates cognitive deficits through the induction of inhibitory synaptic dysfunction in hippocampus (Cai et al., 2022). Inflammatory pain significantly weakens connectivity between the vCA1 and infralimbic cortex (IL) paralleling the onset of cognitive impairments (Shao et al., 2023). Furthermore, CA1 can mediate the comorbidity of neuropathic pain and memory deficits through glutamatergic projections to the mPFC (Han et al., 2023). Other critical limbic components, such as the ACC, are closely associated with processing pain information and decision-making behavior (Davis et al., 1997; Cao et al., 2016). However, the intricate mechanisms linking the amygdala to pain-related cognitive deficits require further investigation.

Even after the noxious stimulus has disappeared, the pain perception persists or intensifies when then pain-inducing external stimulus acts on the body again (Hu et al., 2021; Stegemann et al., 2023). This phenomenon is referred to as pain memory. The formation of memory has been found to impair cognitive function and leads to negative behaviors (Cai et al., 2022; Shackman et al., 2011). In particular, the formation of pain memory can be prevented by restoring E/I balance. This can be achieved by reducing the activity of GABAergic neurons in the MCC (Li et al., 2023) and increasing the activity of the inhibitory neurons in the IC (Xiao et al., 2023).

## 7 Clinical relevance and therapeutic innovations

Chronic pain progression drives functional and structural reorganization within the brain. Gray matter volume decreases occur across distinct cortical regions (Baliki et al., 2012; Obermann et al., 2013), with the magnitude and distribution of atrophy varying according to the intensity and phenotype of persistent pain (Obermann et al., 2013; Wu et al., 2022; Amirmohseni et al., 2016; Alshelh et al., 2018). Significantly increased c-Fos expression is observed in the mPFC of migraine patients (Wu et al., 2022). Patients with chronic trigeminal neuropathic pain exhibit reduced blood oxygenation level dependent (BOLD) signal intensity detected by functional magnetic resonance imaging (fMRI) in the S1 (Obermann et al., 2013), while those with clinical chronic facial pain show decreased T2 signal intensity in the S1 (Alshelh et al., 2018). As pain constitutes a dynamic network imbalance spanning sensory, affective, and cognitive circuits, the disruption of E/I balance maintained by glutamatergic and GABAergic systems serves as the primary mechanism underlying this reorganization (Eto et al., 2012; Watson, 2016; Cao et al., 2019). Research demonstrates that dynamic alterations in glutamate-driven excitatory and GABA-mediated inhibitory neurotransmission are critical determinants of the transition from acute to chronic pain (Bell et al., 2021; Pigott et al., 2023), individual variability in pain sensitivity (Zunhammer et al., 2016; Thiaucourt et al., 2018; Onderwater et al., 2021; Stærmose et al., 2019) and the long-term maintenance of chronic pain states (Zunhammer et al., 2016; Thiaucourt et al., 2018). Notably, distinct patterns of glutamate and GABA changes emerge in the brain, reflecting pathophysiological heterogeneity across pain subtypes (Peek et al., 2020).

Two 7 Tesla magnetic resonance spectroscopy (MRS) studies reveal elevated GABA concentrations in the visual cortex of adult migraineurs during interictal periods (Onderwater et al., 2021)and stable glutamate and glutamine (Glx) levels in the visual cortex across migraine states (Zielman et al., 2017). Additional MRS investigations demonstrate the decreased level of glutamate in sensorimotor and occipital cortex in pediatric patients within 24 h preictally (Cho et al., 2024), a significantly reduced concentration of glutamate in the visual cortex during migraine attacks (Bell et al., 2021) and further declines of glutamate in sensorimotor and occipital cortex after headache attacking postictally (Cho et al., 2024). Notably, higher sensorimotor GABA/Glx ratios correlate with longer disease duration and lower sensorimotor GABA levels associate with shorter time to next migraine attack (Bell et al., 2021). Another separate MRS study indicates negative correlations between Glx levels in the right dorsolateral prefrontal cortex (dlPFC) and migraine severity in episodic migraine without aura patients (Wang et al., 2024).

Other MRS studies across diverse chronic pain conditions reveal converging alterations in glutamate and GABA. Patients with chronic pain syndromes exhibited an increased Glu/GABA ratio in the insula cortex (Thiaucourt et al., 2018). Adolescents with chronic pain shown significantly decreased GABA levels in the left posterior insula, while the Glx level remain unchanged (Pigott et al., 2023). Additionally, elevated levels of circulating soluble interleukin-2 receptor (sCD25) significantly correlate with the Glx/GABA ratio in the ACC, which interplay between peripheral inflammation and central E/I imbalance promotes pain catastrophizing (Huo et al., 2005). Both patients with chronic musculoskeletal pain (Ma J. et al., 2024) and those with primary dysmenorrhea (Chen et al., 2023) demonstrate an increased Glx/GABA ratio in the ACC, whose ratio correlates with comorbid anxiety and depression in chronic pain (Ma J. et al., 2024).

Collectively, these clinical studies demonstrate significant associations between dynamic alterations in glutamate and GABA levels across distinct brain regions and key pain features, including susceptibility to pain attacks, inter-individual variability, and pain persistence (Onderwater et al., 2021; Stærmose et al., 2019). Emerging therapeutic strategies, aimed at restoring cortical E/I balance and directly correcting maladaptive neural circuitry in chronic pain, show promising efficacy. These encompass approaches of traditional Chinese medicine (TCM) and novel non-invasive brain stimulation (NIBS) techniques within chronic pain management frameworks (Pigott et al., 2023; Galafassi et al., 2021; Mawla et al., 2021; Zhang et al., 2025).

Electroacupuncture (EA), a well-established TCM modality for pain relief, demonstrates mechanistic specificity (Mawla et al., 2021; Wei X. Y. et al., 2024; Li R. et al., 2024). Combined resting-state functional MRI (rs-fMRI) and proton magnetic resonance spectroscopy (1H-MRS) evidence reveals that EA ameliorates fibromyalgia pain by indirectly elevating GABA concentrations in the bilateral anterior insula, thereby enhancing functional connectivity between the S1 and bilateral anterior insula regions (Mawla et al., 2021). NIBS techniques, including repetitive transcranial magnetic stimulation (rTMS), transcranial direct current stimulation (tDCS), transcranial alternating current stimulation (tACS), transcranial focused ultrasound (tFUS), transcranial random noise stimulation (tRNS), and temporal interference stimulation (TIS), represent critical alternatives for pharmacotherapy-refractory pain (Jayathilake et al., 2025; Ahn et al., 2019; Liu Y. et al., 2024). They operate by precisely modulating neuroplasticity and restoring E/I balance, while concomitantly exhibiting potential to improve mood and cognition. Recent mechanistic insights show that preemptive tDCS over the M1 attenuates pain-related anxiety via modulation of microstate D-to-E transitions. Preemptive tDCS over dlPFC reduces pain intensity by decreasing microstate D-to-C shifts (Zhang et al., 2025). These innovational treatments improve the precision of pain discrimination and the ability to respond effectively to harmful stimuli, thereby improving pain sensitivity. Concurrently, it can participate in the coding and regulation of pain information, obstruct the downward transmission of sensory signals and induces analgesia, thus rectifying the E/I imbalance and maintaining normal sensory information processing in the cerebral cortex.

## 8 Discussion

The glutamatergic and GABAergic systems play a critical role in maintaining the equilibrium of E/I balance and in the processing of pain within the CNS. The interaction between these two systems determines the encoding and transmission of pain signals. An imbalance in this system can lead to the emergence of a persistent E/I imbalance, enhance nociceptive perception and induce pain-related negative behaviors by affecting the plasticity and stability of neural networks. Current chronic pain treatments often target a single system, aiming to achieve analgesic effects. The direct inhibition of glutamatergic systems has been found to reduce hyperexcitability, including the modulation of glutamate transporter function (Palmer et al., 1994; Nicholls and Attwell, 1990; Gegelashvili and Bjerrum, 2019), glutamate receptor activity (Li et al., 2020) glutamatergic neurons (Kami et al., 2022; Notartomaso et al., 2024) and output neuronal circuits (Hu et al., 2019; Takeda et al., 2022; Cao et al., 2023; Yin et al., 2020; Zhou et al., 2022). Alternatively, the activation of the GABAergic system produces inhibitory effects through the specific activation of GABAergic receptors (Chaparro et al., 2012), neurons (Xiao et al., 2023; Ren et al., 2022; Li et al., 2023) and their output projections (Cao et al., 2023; Wu et al., 2020; Huo et al., 2005; de Andrade et al., 2019; Gao et al., 2023). In particular, the role of the interaction between the glutamatergic and GABAergic systems in pain modulation warrants careful consideration. The specific mechanism underlying this interaction and the potential for exploiting this property in the treatment of chronic pain (Piot et al., 2023; Wen et al., 2022) remain poorly understood. These two systems form a specific neuronal network for pain modulation. The excitatory neurons in the cerebral cortex are regulated artificially to generate inhibitory action through the projection to GABAergic neurons in downward regions, and vice versa. This represents a straightforward unidirectional output mechanism that can be adjusted by excitatory or inhibitory synapses. However, this pathway lacks physiological feedback from the downstream to the upstream. And this can easily result in further degradation and loss of normal function of the downstream normal regions. Once external interventions are removed, a more pronounced disturbance of the E/I balance and associated side effects may occur. Therefore, the simultaneous reconditioning of the two system that have been overly disrupted systems can prevent impairment of physiological function and normalize the perception and E/I balance.

In addition, this review exclusively discusses the output work pattern dominated by the systems for pain processing, without considering the role of the cerebral cortex in the pattern of receiving inputs of pain information. Further research is required to elucidate the manner in which the cerebral cortex detects, classifies and recodes the various components of sensory stimuli. The brainstem, serving as the central hub for descending modulation and endogenous analgesia, plays a critical role in autonomic and affective responses (Gan et al., 2022; Cheriyan and Sheets, 2018; Wu et al., 2020; Huo et al., 2005; de Andrade et al., 2019). It relays preliminarily processed signals to the cerebral cortex, which sorts, integrates, and transmits pain signals back to the brainstem. The brainstem executes pain signaling, initiates endogenous analgesic mechanisms, and further feeds back arousal and affective signals to the cortex, modifying cortical signal encoding processes. Prior research demonstrates that prolonged noxious sensory input induces hyperexcitability of glutamatergic neurons in the ACC. This enhances GABAergic transmission onto dopaminergic (DA) neurons in the VTA, inhibiting DA neurons and producing antidepressant-like behavioral effects. Consequently, this abolishes the “braking” effect of DA transmission within the ACC, leading to sustained hyperactivation of glutamatergic neurons, thereby promoting persistent pain and affective comorbidities. This ACC-VTA-ACC circuit drives a maladaptive sensory-affective interplay and a vicious cycle, perpetuating chronic pain (Song et al., 2024). Furthermore, two neuroimaging studies reveal that during acute pain states, increased PFC-NAc functional connectivity coupled with decreased NAc-mPFC connectivity is predictive of the transition to chronic pain. Conversely, during the emergence of affective pain, enhanced feedback connectivity from the NAc to the mPFC further facilitates pain persistence (Baliki et al., 2012; Park et al., 2022). The specific manifestations of this cortico-brainstem “decision-execution-feedback” signaling circuit under physiological and pathological conditions require further experimental elucidation.

Meanwhile, the circuitries within the CNS may be subject to the influence of a multitude of factors, including inflammation, injury and psychological processes. This can result in complications with regard to the coordination of circuits and presents a challenge in the context of pain medication. Moreover, this review specifically centers on the glutamatergic and GABAergic systems constituting the E/I balance. Other neural types also play significant roles in pain, including monoaminergic neurons (serotonin, norepinephrine, dopamine), peptide-gic neurons (substance P, calcitonin gene-related peptide, opioid peptides), and cholinergic neurons. Further comprehensive reviews are warranted to elucidate the integration and encoding mechanisms of pain signals by these neuronal populations, the dynamic modulation of regional brain function via these neural circuit mechanisms and the therapeutic targeting strategies for developing novel interventions. Similarly, this review primarily addresses interactions between neurons and astrocytes in relation to pain. Other glial cells, notably oligodendrocytes (Kim and Angulo, 2025; Li D. et al., 2024; Iwata et al., 2024) and microglia (Kohno and Tsuda, 2025; Malcangio and Sideris-Lampretsas, 2025), also engage in crucial bidirectional interactions with neurons. These interactions which are also the key determinants of neuronal excitability, are intricately linked to neuronal development, myelination, and synaptic plasticity, and thus warrant focused investigation in future pain research.

Pain represents a dynamic network imbalance involving sensory, affective, and cognitive circuits. Current mechanistic investigations remain largely confined to rodent-based animal studies. Research in rodent models offers unique advantages for dissecting molecular and cellular mechanisms, permitting invasive genetic manipulations. Through optogenetic and chemogenetic modulation of specific neurons or neural circuits can be manipulated to verify the micro-scale mechanisms and facilitate high-throughput drug screening. However, rodent studies cannot fully recapitulate the affective-cognitive dimensions of human pain, particularly in modeling affective-cognitive comorbidities. The cross-species validation integrating rodents, non-human primates and human models is essential to delineate evolutionary conservation and species-specific divergence in nociceptive pathways. This approach comprehensively elucidates the multidimensional characteristics of pain—spanning sensory, affective, and cognitive domains. The study on non-human primate can bridge this translational gap through their high similarity to humans in neuroanatomical connectivity and advanced behavioral repertoires. NHPs effectively model affective integration of pain and reveal its impact on cognition, directly predicting clinical drug efficacy and validating neuromodulatory mechanisms (Shackman et al., 2011). Human researches constitute the cornerstone for clinical phenotyping and individualized mechanistic profiling. Direct assessment of diverse pain phenotypes in humans enables precision-targeted interventions, guiding tailored therapeutic strategies.

Our analysis highlights a significant positive correlation between elevated glutamate/GABA ratios and pain sensitization. Contemporary studies employ diverse innovative methodologies, such as high-performance liquid chromatography (HPLC) for ex vivo quantification (Cseh et al., 2020), photosensitive photopharmacology for spatiotemporal receptor manipulation (Notartomaso et al., 2024), whole-cell patch-clamp electrophysiology for single-neuron excitability profiling (Tullis et al., 2023; Liu W. et al., 2024), two-photon imaging combined with calcium indicators for circuit-level activity mapping (Guo et al., 2023; Tullis et al., 2023; Liu W. et al., 2024; Zhang et al., 2023), MRS for *in vivo* neurochemical measurement (Onderwater et al., 2021; Zielman et al., 2017) and fMRI for network connectivity analysis (Mawla et al., 2021). These creative methods are adopted to assess functional alterations in glutamatergic and GABAergic systems, including neurotransmitter concentration shifts, receptor functional dynamics, neuronal excitability changes, and migration of activity in brain regions. Despite substantial advances in mechanistic understanding, key limitations remain. Chronic pain progression involves dynamic co-evolution of glutamatergic and GABAergic systems across extended timescales. Current technologies cannot longitudinally capture multi-transmitter interactions with simultaneous cellular resolution, hindering elucidation compensatory plasticity between excitatory and inhibitory circuits, temporal coordination of neuromodulator release events, and system-wide adaptation thresholds driving pain chronification. Meanwhile, it is also difficult to present time points at which the multi-dimensional pain experiences occur and how these multi-dimensional experiences influence each other. Notably, species differences between rodents and humans result in disparities in the practicality and applicability of technical approaches, posing a significant barrier to the clinical translation of current research findings.

Concurrently, the inherent constraints of single-modality detection techniques hinder the capture of multifaceted characteristics of chronic pain. Multimodal neuroimaging studies enable a comprehensive assessment of cortical excitability changes and facilitates precise targeting of therapeutic interventions (Widman et al., 2025; Quidé et al., 2024; Zhang et al., 2024; Namgung et al., 2025). Consequently, future research must prioritize the integration of cross-species validation with multimodal imaging technologies to analyze critical transition points in the brain from acute to chronic pain (Baliki et al., 2012; Park et al., 2022), to decipher dynamic multidimensional shifts spanning sensory perception, affect, and cognition in the pain experience, and to investigate individual differences in pain sensitivity and underlying genetic susceptibility. Furthermore, leveraging artificial intelligence to co-develop diagnostic and progression models for pain is crucial. These models should guide the timing of therapeutic interventions and optimize neuromodulation parameters for non-invasive neurostimulation therapies, marking a critical direction for future advancements in the field.

## 9 Conclusion

The cerebral cortex plays a complex role in the processing of pain information, involving a number of different processes, including neuronal responses in individual cortical regions, neuron-astrocytes interaction, the sharing and cascading of inter-cortical signal and downward cortical modulation. Collectively, these processes form a complex pain modulation network that controls the propagation and return of pain information in the brain. However, the modulation network is disrupted in individual with chronic pain, and this disruption is strongly associated with the progression of chronic pain. The onset and persistence of chronic pain is closely linked to an E/I imbalance between glutamatergic and GABAergic systems within the cerebral cortex. This imbalance leads to destabilization of neural networks, the biased output of information and the manifestation of abnormal pain perception. The interaction between neurons and astrocytes is disrupted, which can exacerbate the E/I disequilibrium in the brain, further interfering with the processing of pain signals and potentially leading to excitatory neurotoxicity. The process in sharing and cascading of inter-cortical signal influences how individuals cope with pain and their emotional state and contributes to the pain chronicity. The cerebral cortex converts sensory signals to perceptual signals and causes changes in plasticity functional and structural plasticity through modulation of downstream brain regions. Meanwhile, future studies need to further explore the mechanisms of interaction between these systems and develop more effective treatments to restore the excitatory-inhibitory balance in the cerebral cortex and improve the symptoms of chronic pain patients. This is due to the importance of interactions between glutamatergic and GABAergic systems.

## References

[ref1] Ahn S. Prim J. H. Alexander M. L. McCulloch K. L. Fröhlich F. (2019). Identifying and engaging neuronal oscillations by transcranial alternating current stimulation in patients with chronic low back pain: a randomized, crossover, double-blind, sham-controlled pilot study. J. Pain 20:277. doi: 10.1016/j.jpain.2018.09.004 PMC6382517 30268803

[ref2] Alshelh Z. Di Pietro F. Mills E. P. Vickers E. R. Peck C. C. Murray G. M.. (2018). Altered regional brain T2 relaxation times in individuals with chronic orofacial neuropathic pain. Neuroimage Clin. 19, 167–173. doi: 10.1016/j.nicl.2018.04.015, PMID: 30035014 PMC6051476

[ref3] Amirmohseni S. Segelcke D. Reichl S. Wachsmuth L. Görlich D. Faber C.. (2016). Characterization of incisional and inflammatory pain in rats using functional tools of MRI. NeuroImage 127, 110–122. doi: 10.1016/j.neuroimage.2015.11.052, PMID: 26631818

[ref4] Araque A. Parpura V. Sanzgiri R. P. Haydon P. G. (1999). Tripartite synapses: glia, the unacknowledged partner. Trends Neurosci. 22, 208–215. doi: 10.1016/S0166-2236(98)01349-6, PMID: 10322493

[ref5] Baliki M. N. Petre B. Torbey S. Herrmann K. M. Huang L. Schnitzer T. J.. (2012). Corticostriatal functional connectivity predicts transition to chronic back pain. Nat. Neurosci. 15, 1117–1119. doi: 10.1038/nn.3153, PMID: 22751038 PMC3411898

[ref6] Bell T. Stokoe M. Khaira A. Webb M. Noel M. Amoozegar F.. (2021). GABA and glutamate in pediatric migraine. Pain 162, 300–308. doi: 10.1097/j.pain.0000000000002022, PMID: 33326202 PMC7737876

[ref7] Benson C. Mifflin K. Kerr B. Jesudasan S. J. Dursun S. Baker G. (2015). Biogenic amines and the amino acids GABA and glutamate: relationships with pain and depression. Mod. Trends Pharmacopsychiatry 30, 67–79. doi: 10.1159/000435933 26437459

[ref8] Bhatt R. R. Haddad E. Zhu A. H. Thompson P. M. Gupta A. Mayer E. A.. (2023). Mapping brain structure variability in chronic pain: the role of widespreadness and pain type and its mediating relationship with suicide attempt. Biol. Psychiatry 95, 473–481. doi: 10.1016/j.biopsych.2023.07.016 37543299 PMC10838358

[ref9] Botvinik-Nezer R. Petre B. Ceko M. Lindquist M. A. Friedman N. P. Wager T. D. (2024). Placebo treatment affects brain systems related to affective and cognitive processes, but not nociceptive pain. Nat. Commun. 15:6017. doi: 10.1038/s41467-024-50103-8, PMID: 39019888 PMC11255344

[ref10] Buetfering C. Zhang Z. Pitsiani M. Smallridge J. Boven E. McElligott S.. (2022). Behaviorally relevant decision coding in primary somatosensory cortex neurons. Nat. Neurosci. 25, 1225–1236. doi: 10.1038/s41593-022-01151-0, PMID: 36042310 PMC7613627

[ref11] Cahill M. K. Collard M. Tse V. Reitman M. E. Etchenique R. Kirst C.. (2024). Network-level encoding of local neurotransmitters in cortical astrocytes. Nature 629, 146–153. doi: 10.1038/s41586-024-07311-5, PMID: 38632406 PMC11062919

[ref12] Cai X. Qiu L. Wang C. Yang H. Zhou Z. Mao M.. (2022). Hippocampal inhibitory synapsis deficits induced by α5-containing GABA(a) receptors mediate chronic neuropathic pain-related cognitive impairment. Mol. Neurobiol. 59, 6049–6061. doi: 10.1007/s12035-022-02955-8, PMID: 35849280

[ref13] Cao B. Wang J. Mu L. Poon D. C. Li Y. (2016). Impairment of decision making associated with disruption of phase-locking in the anterior cingulate cortex in viscerally hypersensitive rats. Exp. Neurol. 286, 21–31. doi: 10.1016/j.expneurol.2016.09.010, PMID: 27664369

[ref14] Cao F. L. Xu M. Gong K. Wang Y. Wang R. Chen X.. (2019). Imbalance between excitatory and inhibitory synaptic transmission in the primary somatosensory cortex caused by persistent nociception in rats. J. Pain 20, 917–931. doi: 10.1016/j.jpain.2018.11.014, PMID: 30742914

[ref15] Cao P. Zhang M. Ni Z. Song X. J. Yang C. L. Mao Y.. (2023). Green light induces antinociception via visual-somatosensory circuits. Cell Rep. 42:112290. doi: 10.1016/j.celrep.2023.112290, PMID: 36947545

[ref16] Cardoso-Cruz H. Laranjeira I. Monteiro C. Galhardo V. (2022). Altered prefrontal-striatal theta-band oscillatory dynamics underlie working memory deficits in neuropathic pain rats. Eur. J. Pain 26, 1546–1568. doi: 10.1002/ejp.1982, PMID: 35603472

[ref17] Cardoso-Cruz H. Lima D. Galhardo V. (2013a). Impaired spatial memory performance in a rat model of neuropathic pain is associated with reduced hippocampus-prefrontal cortex connectivity. J. Neurosci. 33, 2465–2480. doi: 10.1523/JNEUROSCI.5197-12.2013, PMID: 23392675 PMC6619155

[ref18] Cardoso-Cruz H. Paiva P. Monteiro C. Galhardo V. (2019). Bidirectional optogenetic modulation of prefrontal-hippocampal connectivity in pain-related working memory deficits. Sci. Rep. 9:10980. doi: 10.1038/s41598-019-47555-0, PMID: 31358862 PMC6662802

[ref19] Cardoso-Cruz H. Sousa M. Vieira J. B. Lima D. Galhardo V. (2013b). Prefrontal cortex and mediodorsal thalamus reduced connectivity is associated with spatial working memory impairment in rats with inflammatory pain. Pain 154, 2397–2406. doi: 10.1016/j.pain.2013.07.020, PMID: 23872106

[ref20] Chang C. L. Trimbuch T. Chao H. T. Jordan J. C. Herman M. A. Rosenmund C. (2014). Investigation of synapse formation and function in a glutamatergic-GABAergic two-neuron microcircuit. J. Neurosci. 34, 855–868. doi: 10.1523/JNEUROSCI.0229-13.2014, PMID: 24431444 PMC6608345

[ref21] Chaparro L. E. Wiffen P. J. Moore R. A. Gilron I. (2012). Combination pharmacotherapy for the treatment of neuropathic pain in adults. Cochrane Database Syst. Rev. 2020, 230–251. doi: 10.1002/14651858.CD008943.pub2, PMID: 22786518 PMC6481651

[ref22] Chen X. Huang Z. Wu X. Han S. Wu P. Li Y. (2023). Assessment of neurotransmitter imbalances within the anterior cingulate cortex in women with primary dysmenorrhea: An initial proton magnetic resonance spectroscopy study. Eur. J. Radiol. 167:111079. doi: 10.1016/j.ejrad.2023.111079, PMID: 37683332

[ref23] Chen L. Li X. Tjia M. Thapliyal S. (2022). Homeostatic plasticity and excitation-inhibition balance: the good, the bad, and the ugly. Curr. Opin. Neurobiol. 75:102553. doi: 10.1016/j.conb.2022.102553, PMID: 35594578 PMC9477500

[ref24] Chen C. Niehaus J. K. Dinc F. Huang K. L. Barnette A. L. Tassou A.. (2024). Neural circuit basis of placebo pain relief. Nature 632, 1092–1100. doi: 10.1038/s41586-024-07816-z, PMID: 39048016 PMC11358037

[ref25] Chen T. Wang W. Dong Y. L. Zhang M. M. Wang J. Koga K.. (2014). Postsynaptic insertion of AMPA receptor onto cortical pyramidal neurons in the anterior cingulate cortex after peripheral nerve injury. Mol. Brain 7:76. doi: 10.1186/s13041-014-0076-8, PMID: 25359681 PMC4221704

[ref26] Cheng Y. T. Luna-Figueroa E. Woo J. Chen H. C. Lee Z. F. Harmanci A. S.. (2023). Inhibitory input directs astrocyte morphogenesis through glial GABA(B)R. Nature 617, 369–376. doi: 10.1038/s41586-023-06010-x, PMID: 37100909 PMC10733939

[ref27] Cheriyan J. Sheets P. L. (2018). Altered excitability and local connectivity of mPFC-PAG neurons in a mouse model of neuropathic pain. J. Neurosci. 38, 4829–4839. doi: 10.1523/JNEUROSCI.2731-17.2018, PMID: 29695413 PMC6596022

[ref28] Cheriyan J. Sheets P. L. (2020). Peripheral nerve injury reduces the excitation-inhibition balance of basolateral amygdala inputs to prelimbic pyramidal neurons projecting to the periaqueductal gray. Mol. Brain 13:100. doi: 10.1186/s13041-020-00638-w, PMID: 32600466 PMC7325111

[ref29] Cho L. Y. Bell T. K. Craddock L. Godfrey K. J. Hershey A. D. Kuziek J.. (2024). Region-specific changes in brain glutamate and gamma-aminobutyric acid across the migraine attack in children and adolescents. Pain 165, 2749–2761. doi: 10.1097/j.pain.0000000000003289, PMID: 38833578 PMC11562757

[ref30] Comitato A. Bardoni R. (2021). Presynaptic inhibition of pain and touch in the spinal cord: from receptors to circuits. Int. J. Mol. Sci. 22:414. doi: 10.3390/ijms22010414, PMID: 33401784 PMC7795800

[ref31] Cseh E. K. Veres G. Körtési T. Polyák H. Nánási N. Tajti J.. (2020). Neurotransmitter and tryptophan metabolite concentration changes in the complete Freund's adjuvant model of orofacial pain. J. Headache Pain 21:35. doi: 10.1186/s10194-020-01105-6, PMID: 32316909 PMC7175490

[ref32] Cui L. Guo J. Cranfill S. L. Gautam M. Bhattarai J. Olson W.. (2022). Glutamate in primary afferents is required for itch transmission. Neuron 110, 809–823. doi: 10.1016/j.neuron.2021.12.007, PMID: 34986325 PMC8898340

[ref33] Danjo Y. Shigetomi E. Hirayama Y. J. Kobayashi K. Ishikawa T. Fukazawa Y.. (2022). Transient astrocytic mGluR5 expression drives synaptic plasticity and subsequent chronic pain in mice. J. Exp. Med. 219:989. doi: 10.1084/jem.20210989, PMID: 35319723 PMC8952801

[ref34] Davis K. D. Taylor S. J. Crawley A. P. Wood M. L. Mikulis D. J. (1997). Functional MRI of pain- and attention-related activations in the human cingulate cortex. J. Neurophysiol. 77, 3370–3380. doi: 10.1152/jn.1997.77.6.3370, PMID: 9212281

[ref35] de Andrade E. M. Martinez R. C. R. Pagano R. L. Lopes P. S. S. Auada A. V. V. Gouveia F. V.. (2019). Neurochemical effects of motor cortex stimulation in the periaqueductal gray during neuropathic pain. J. Neurosurg. 132, 239–251. doi: 10.3171/2018.7.JNS173239, PMID: 30611141

[ref36] de Ceglia R. Ledonne A. Litvin D. G. Lind B. L. Carriero G. Latagliata E. C.. (2023). Specialized astrocytes mediate glutamatergic gliotransmission in the CNS. Nature 622, 120–129. doi: 10.1038/s41586-023-06502-w, PMID: 37674083 PMC10550825

[ref37] Eto K. Ishibashi H. Yoshimura T. Watanabe M. Miyamoto A. Ikenaka K.. (2012). Enhanced GABAergic activity in the mouse primary somatosensory cortex is insufficient to alleviate chronic pain behavior with reduced expression of neuronal potassium-chloride cotransporter. J. Neurosci. 32, 16552–16559. doi: 10.1523/JNEUROSCI.2104-12.2012, PMID: 23175811 PMC6621771

[ref38] Falconi-Sobrinho L. L. Dos Anjos-Garcia T. Elias-Filho D. H. Coimbra N. C. (2017). Unravelling cortico-hypothalamic pathways regulating unconditioned fear-induced antinociception and defensive behaviours. Neuropharmacology 113, 367–385. doi: 10.1016/j.neuropharm.2016.10.001, PMID: 27717879

[ref39] Fan Y. F. Guan S. Y. Luo L. Li Y. J. Yang L. Zhou X. X.. (2018). Tetrahydroxystilbene glucoside relieves the chronic inflammatory pain by inhibiting neuronal apoptosis, microglia activation, and GluN2B overexpression in anterior cingulate cortex. Mol. Pain 14:1744806918814367. doi: 10.1177/1744806918814367, PMID: 30380983 PMC6259074

[ref40] Ferguson B. R. Gao W. J. (2018). PV interneurons: critical regulators of E/I balance for prefrontal cortex-dependent behavior and psychiatric disorders. Front Neural Circuits. 12:37. doi: 10.3389/fncir.2018.00037, PMID: 29867371 PMC5964203

[ref41] Fischer B. D. (2017). GABA(a) receptors as targets for the Management of Pain-related Disorders: historical perspective and update. CNS Neurol. Disord. Drug Targets 16, 658–663. doi: 10.2174/1871527316666170207155149, PMID: 28176641

[ref42] Galafassi G. Z. Pires S. de Aguiar P. H. Simm R. F. Franceschini P. R. Filho M. P.. (2021). Neuromodulation for medically refractory neuropathic pain: spinal cord stimulation, deep brain stimulation, motor cortex stimulation, and posterior insula stimulation. World Neurosurg. 146, 246–260. doi: 10.1016/j.wneu.2020.11.048, PMID: 33217591

[ref43] Gan Z. Gangadharan V. Liu S. Körber C. Tan L. L. Li H.. (2022). Layer-specific pain relief pathways originating from primary motor cortex. Science 378, 1336–1343. doi: 10.1126/science.add4391, PMID: 36548429

[ref44] Ganguly K. Schinder A. F. Wong S. T. Poo M. (2001). GABA itself promotes the developmental switch of neuronal GABAergic responses from excitation to inhibition. Cell 105, 521–532. doi: 10.1016/S0092-8674(01)00341-5, PMID: 11371348

[ref45] Gao F. Huang J. Huang G. B. You Q. L. Yao S. Zhao S. T.. (2023). Elevated prelimbic cortex-to-basolateral amygdala circuit activity mediates comorbid anxiety-like behaviors associated with chronic pain. J. Clin. Invest. 133:356. doi: 10.1172/JCI166356, PMID: 36917193 PMC10145931

[ref46] Garcia-Larrea L. Peyron R. (2013). Pain matrices and neuropathic pain matrices: a review. Pain 154, S29–S43. doi: 10.1016/j.pain.2013.09.001 24021862

[ref47] Gegelashvili G. Bjerrum O. J. (2019). Glutamate transport system as a key constituent of glutamosome: molecular pathology and pharmacological modulation in chronic pain. Neuropharmacology 161:107623. doi: 10.1016/j.neuropharm.2019.04.029, PMID: 31047920

[ref48] Gnall K. E. Jochimsen K. N. Brewer J. R. Bakhshaie J. Vranceanu A. M. (2025). Pain catastrophizing and pain anxiety mediate changes in physical function in a mind-body intervention for adults with traumatic orthopedic injuries. Pain 166, 1418–1424. doi: 10.1097/j.pain.0000000000003477, PMID: 39661363 PMC12188628

[ref49] Guida F. Luongo L. Marmo F. Romano R. Iannotta M. Napolitano F.. (2015). Palmitoylethanolamide reduces pain-related behaviors and restores glutamatergic synapses homeostasis in the medial prefrontal cortex of neuropathic mice. Mol. Brain 8:47. doi: 10.1186/s13041-015-0139-5, PMID: 26260027 PMC4532244

[ref50] Guo F. Lin S. D. Du Y. Hu T. T. Wang Y. Chen Z.. (2023). Secondary somatosensory cortex glutamatergic innervation of the thalamus facilitates pain. Pain 165, 1142–1153. doi: 10.1097/j.pain.0000000000003117, PMID: 38112733

[ref51] Han S. Ren J. Li Z. Wen J. Jiang B. Wei X. (2023). Deactivation of dorsal CA1 pyramidal neurons projecting to medial prefrontal cortex contributes to neuropathic pain and short-term memory impairment. Pain 165:3100. doi: 10.1097/j.pain.0000000000003100, PMID: 37889600

[ref52] Hogrefe N. Blom S. M. Valentinova K. Ntamati N. R. Jonker L. J. E. Nevian N. E.. (2022). Long-lasting, pathway-specific impairment of a novel form of spike-timing-dependent Long-term depression by neuropathic pain in the anterior cingulate cortex. J. Neurosci. 42, 2166–2179. doi: 10.1523/JNEUROSCI.0326-21.2022, PMID: 35078926 PMC8936394

[ref53] Hu T. T. Wang R. R. Du Y. Guo F. Wu Y. X. Wang Y.. (2019). Activation of the intrinsic pain inhibitory circuit from the Midcingulate Cg2 to zona Incerta alleviates neuropathic pain. J. Neurosci. 39, 9130–9144. doi: 10.1523/JNEUROSCI.1683-19.2019, PMID: 31604834 PMC6855685

[ref54] Hu S. W. Zhang Q. Xia S. H. Zhao W. N. Li Q. Z. Yang J. X.. (2021). Contralateral projection of anterior cingulate cortex contributes to Mirror-image pain. J. Neurosci. 41, 9988–10003. doi: 10.1523/JNEUROSCI.0881-21.2021, PMID: 34642215 PMC8638682

[ref55] Huo F. Q. Wang J. Li Y. Q. Chen T. Han F. Tang J. S. (2005). Gabaergic neurons express mu-opioid receptors in the ventrolateral orbital cortex of the rat. Neurosci. Lett. 382, 265–268. doi: 10.1016/j.neulet.2005.03.070, PMID: 15899549

[ref56] Iqbal Z. Liu S. Lei Z. Ramkrishnan A. S. Akter M. Li Y. (2022). Astrocyte l-lactate signaling in the ACC regulates visceral pain aversive memory in rats. Cells 12:26. doi: 10.3390/cells12010026, PMID: 36611820 PMC9818423

[ref57] Ishikawa T. Murata K. Okuda H. Potapenko I. Hori K. Furuyama T.. (2023). Pain-related neuronal ensembles in the primary somatosensory cortex contribute to hyperalgesia and anxiety. iScience. 26:106332. doi: 10.1016/j.isci.2023.106332, PMID: 36968067 PMC10033994

[ref58] Islam J. Rahman M. T. Ali M. Kim H. K. Kc E. Park Y. S. (2025). Optogenetic inhibition of ventrolateral orbitofrontal cortex astrocytes facilitates ventrolateral periaqueductal gray glutamatergic activity to reduce hypersensitivity in infraorbital nerve injury rat model. J. Headache Pain 26:41. doi: 10.1186/s10194-025-01977-6, PMID: 39994518 PMC11854010

[ref59] Iwata K. Hayashi Y. Hitomi S. Tsuboi Y. Shinoda M. (2024). Non-neuronal cells act as crucial players in neuropathic orofacial pain. J. Oral Biosci. 66, 491–495. doi: 10.1016/j.job.2024.07.005, PMID: 39032826

[ref60] Jasmin L. Rabkin S. D. Granato A. Boudah A. Ohara P. T. (2003). Analgesia and hyperalgesia from GABA-mediated modulation of the cerebral cortex. Nature 424, 316–320. doi: 10.1038/nature01808, PMID: 12867983

[ref61] Jayathilake N. J. Phan T. T. Kim J. Lee K. P. Park J. M. (2025). Modulating neuroplasticity for chronic pain relief: noninvasive neuromodulation as a promising approach. Exp. Mol. Med. 57, 501–514. doi: 10.1038/s12276-025-01409-0, PMID: 40025172 PMC11958754

[ref62] Jee H. J. Zhu E. Sun M. Liu W. Zhang Q. Wang J. (2023). Anterior cingulate cortex regulates pain catastrophizing-like behaviors in rats. Mol. Brain 16:71. doi: 10.1186/s13041-023-01060-8, PMID: 37833814 PMC10576271

[ref63] Ji G. Neugebauer V. (2011). Pain-related deactivation of medial prefrontal cortical neurons involves mGluR1 and GABA(a) receptors. J. Neurophysiol. 106, 2642–2652. doi: 10.1152/jn.00461.2011, PMID: 21880942 PMC3214095

[ref64] Ji G. Sun H. Fu Y. Li Z. Pais-Vieira M. Galhardo V.. (2010). Cognitive impairment in pain through amygdala-driven prefrontal cortical deactivation. J. Neurosci. 30, 5451–5464. doi: 10.1523/JNEUROSCI.0225-10.2010, PMID: 20392966 PMC2868074

[ref65] Jiang W. Gong M. Shen L. Yu C. Ruan H. Chen P.. (2025). The receptor for advanced glycation end-products in the mouse anterior cingulate cortex is involved in neuron–astrocyte coupling in chronic inflammatory pain and anxiety comorbidity. Mol. Neurobiol. 62, 7183–7204. doi: 10.1007/s12035-025-04713-y, PMID: 39863743 PMC12078453

[ref66] Jin Y. Meng Q. Mei L. Zhou W. Zhu X. Mao Y.. (2020). A somatosensory cortex input to the caudal dorsolateral striatum controls comorbid anxiety in persistent pain. Pain 161, 416–428. doi: 10.1097/j.pain.0000000000001724, PMID: 31651582

[ref67] Jones A. F. Sheets P. L. (2020). Sex-specific disruption of distinct mPFC inhibitory neurons in spared-nerve injury model of neuropathic pain. Cell Rep. 31:107729. doi: 10.1016/j.celrep.2020.107729, PMID: 32521254 PMC7372908

[ref68] Kami K. Tajima F. Senba E. (2022). Brain mechanisms of exercise-induced Hypoalgesia: to find a way out from "fear-avoidance belief". Int. J. Mol. Sci. 23:886. doi: 10.3390/ijms23052886, PMID: 35270027 PMC8911154

[ref69] Kang D. Hesam-Shariati N. McAuley J. H. Alam M. Trost Z. Rae C. D.. (2021). Disruption to normal excitatory and inhibitory function within the medial prefrontal cortex in people with chronic pain. Eur. J. Pain 25, 2242–2256. doi: 10.1002/ejp.1838, PMID: 34242465

[ref70] Kiani F. A. Li H. Nan S. Li Q. Lei Q. Yin R.. (2024). Electroacupuncture relieves neuropathic pain via adenosine 3 receptor activation in the spinal cord dorsal horn of mice. Int. J. Mol. Sci. 25:242. doi: 10.3390/ijms251910242, PMID: 39408573 PMC11475944

[ref71] Kim W. Angulo M. C. (2025). Unraveling the role of oligodendrocytes and myelin in pain. J. Neurochem. 169:e16206. doi: 10.1111/jnc.16206, PMID: 39162089 PMC11657919

[ref72] Kim S. K. Hayashi H. Ishikawa T. Shibata K. Shigetomi E. Shinozaki Y.. (2016). Cortical astrocytes rewire somatosensory cortical circuits for peripheral neuropathic pain. J. Clin. Invest. 126, 1983–1997. doi: 10.1172/JCI82859, PMID: 27064281 PMC4855913

[ref73] Kim S. K. Kato G. Ishikawa T. Nabekura J. (2011). Phase-specific plasticity of synaptic structures in the somatosensory cortex of living mice during neuropathic pain. Mol. Pain 7:87. doi: 10.1186/1744-8069-7-87, PMID: 22067412 PMC3223139

[ref74] Kim W. Kim S. K. Nabekura J. (2017). Functional and structural plasticity in the primary somatosensory cortex associated with chronic pain. J. Neurochem. 141, 499–506. doi: 10.1111/jnc.14012, PMID: 28278355

[ref75] Kim H. R. Long M. Sekerková G. Maes A. Kennedy A. Martina M. (2024). Hypernegative GABA(a) reversal potential in pyramidal cells contributes to medial prefrontal cortex deactivation in a mouse model of neuropathic pain. J. Pain 25, 522–532. doi: 10.1016/j.jpain.2023.09.021, PMID: 37793537 PMC10841847

[ref76] Koga K. Shimoyama S. Yamada A. Furukawa T. Nikaido Y. Furue H.. (2018). Chronic inflammatory pain induced GABAergic synaptic plasticity in the adult mouse anterior cingulate cortex. Mol. Pain 14:1744806918783478. doi: 10.1177/1744806918783478, PMID: 29956582 PMC6096674

[ref77] Koh W. Kwak H. Cheong E. Lee C. J. (2023). GABA tone regulation and its cognitive functions in the brain. Nat. Rev. Neurosci. 24, 523–539. doi: 10.1038/s41583-023-00724-7, PMID: 37495761

[ref78] Kohno K. Tsuda M. (2025). Neuron-microglia interactions modulating neuropathic pain. Int. Immunol. 2:dxaf022. doi: 10.1093/intimm/dxaf022, PMID: 40251994

[ref79] Korkut S. (2025). Comparison of the predictive role of spiritual well-being and pain intensity on pain catastrophizing in acute and chronic pain. Pain Manag. Nurs. 26, e254–e260. doi: 10.1016/j.pmn.2024.12.024, PMID: 39848812

[ref80] Kragel P. A. Kano M. Van Oudenhove L. Ly H. G. Dupont P. Rubio A.. (2018). Generalizable representations of pain, cognitive control, and negative emotion in medial frontal cortex. Nat. Neurosci. 21, 283–289. doi: 10.1038/s41593-017-0051-7, PMID: 29292378 PMC5801068

[ref81] Kuner R. Kuner T. (2021). Cellular circuits in the brain and their modulation in acute and chronic pain. Physiol. Rev. 101, 213–258. doi: 10.1152/physrev.00040.2019, PMID: 32525759

[ref82] Lee H. G. Wheeler M. A. Quintana F. J. (2022). Function and therapeutic value of astrocytes in neurological diseases. Nat. Rev. Drug Discov. 21, 339–358. doi: 10.1038/s41573-022-00390-x, PMID: 35173313 PMC9081171

[ref83] Li C. Lei Y. Tian Y. Xu S. Shen X. Wu H.. (2019). The etiological contribution of GABAergic plasticity to the pathogenesis of neuropathic pain. Mol. Pain 15:1744806919847366. doi: 10.1177/1744806919847366, PMID: 30977423 PMC6509976

[ref84] Li X. H. Shi W. Chen Q. Y. Hao S. Miao H. H. Miao Z.. (2023). Activation of the glutamatergic cingulate cortical-cortical connection facilitates pain in adult mice. Commun. Biol. 6:1247. doi: 10.1038/s42003-023-05589-1, PMID: 38071375 PMC10710420

[ref85] Li R. Sun J. Luo K. Luo N. Sun R. Gao F.. (2024). Electroacupuncture and carbamazepine for patients with trigeminal neuralgia: a randomized, controlled, 2 × 2 factorial trial. J. Neurol. 271, 5122–5136. doi: 10.1007/s00415-024-12433-x, PMID: 38816482 PMC11319385

[ref86] Li D. Yang K. Li J. Xu X. Gong L. Yue S.. (2024). Single-cell sequencing reveals glial cell involvement in development of neuropathic pain via myelin sheath lesion formation in the spinal cord. J. Neuroinflammation 21:213. doi: 10.1186/s12974-024-03207-3, PMID: 39217340 PMC11365210

[ref87] Li H. Zhan X. Zhao X. Zhou J. Chen K. Chen Y.. (2025). Exploring the differences in resting state functional magnetic resonance imaging brain activity in patients with chronic low back pain based on ALE meta-analysis. J. Pain 32:105442. doi: 10.1016/j.jpain.2025.105442, PMID: 40403861

[ref88] Li J. Zhang L. Xu C. Lin Y. H. Zhang Y. Wu H. Y.. (2020). Prolonged use of NMDAR antagonist develops analgesic tolerance in neuropathic pain via nitric oxide reduction-induced GABAergic disinhibition. Neurotherapeutics 17, 1016–1030. doi: 10.1007/s13311-020-00883-w, PMID: 32632774 PMC7609518

[ref89] Li M. Zhou H. Teng S. Yang G. (2022). Activation of VIP interneurons in the prefrontal cortex ameliorates neuropathic pain aversiveness. Cell Rep. 40:111333. doi: 10.1016/j.celrep.2022.111333, PMID: 36103825 PMC9520588

[ref90] Li X. Zhu Y. Sun H. Shen Z. Sun J. Xiao S.. (2023). Electroacupuncture inhibits pain memory and related anxiety-like behaviors by blockading the GABA(B) receptor function in the Midcingulate cortex. Mol. Neurobiol. 60, 6613–6626. doi: 10.1007/s12035-023-03467-9, PMID: 37468738 PMC10533721

[ref91] Liu W. Chen Q. Y. Li X. H. Zhou Z. Zhuo M. (2024). Cortical tagged synaptic Long-term depression in the anterior cingulate cortex of adult mice. J. Neurosci. 44:e0028242024. doi: 10.1523/JNEUROSCI.0028-24.2024, PMID: 39054067 PMC11358531

[ref92] Liu Y. Sun J. Wu C. Ren J. He Y. Sun N.. (2024). Characterizing the opioidergic mechanisms of repetitive transcranial magnetic stimulation-induced analgesia: a randomized controlled trial. Pain 165, 2035–2043. doi: 10.1097/j.pain.0000000000003220, PMID: 38537053 PMC11331833

[ref93] Liu S. B. Zhang M. M. Cheng L. F. Shi J. Lu J. S. Zhuo M. (2015). Long-term upregulation of cortical glutamatergic AMPA receptors in a mouse model of chronic visceral pain. Mol. Brain 8:76. doi: 10.1186/s13041-015-0169-z, PMID: 26585043 PMC4653882

[ref94] López-Avila A. Coffeen U. Ortega-Legaspi J. M. del Angel R. Pellicer F. (2004). Dopamine and NMDA systems modulate long-term nociception in the rat anterior cingulate cortex. Pain 111, 136–143. doi: 10.1016/j.pain.2004.06.010, PMID: 15327817

[ref95] Lucas J. M. Ji Y. Masri R. (2011). Motor cortex stimulation reduces hyperalgesia in an animal model of central pain. Pain 152, 1398–1407. doi: 10.1016/j.pain.2011.02.025, PMID: 21396776 PMC3098950

[ref96] Lyu Z. Guo Y. Gong Y. Fan W. Dou B. Li N.. (2021). The role of neuroglial crosstalk and synaptic plasticity-mediated central sensitization in acupuncture analgesia. Neural Plast. 2021, 1–18. doi: 10.1155/2021/8881557, PMID: 33531894 PMC7834789

[ref97] Ma J. Subramaniam P. Yancey J. R. Farrington A. A. McGlade E. C. Renshaw P. F.. (2024). Elevated circulating soluble interleukin-2 receptor (sCD25) level is associated with prefrontal excitatory-inhibitory imbalance in individuals with chronic pain: a proton MRS study. Brain Behav. Immun. 120, 1–9. doi: 10.1016/j.bbi.2024.05.020, PMID: 38772429 PMC11269041

[ref98] Ma L. Yue L. Liu S. Xu S. Tong J. Sun X.. (2024). A distinct neuronal ensemble of prelimbic cortex mediates spontaneous pain in rats with peripheral inflammation. Nat. Commun. 15:7922. doi: 10.1038/s41467-024-52243-3, PMID: 39256428 PMC11387830

[ref99] Malcangio M. Sideris-Lampretsas G. (2025). How microglia contribute to the induction and maintenance of neuropathic pain. Nat. Rev. Neurosci. 26, 263–275. doi: 10.1038/s41583-025-00914-5, PMID: 40128335

[ref100] Mao X. Cai D. Lou W. (2022). Music alleviates pain perception in depression mouse models by promoting the release of glutamate in the hippocampus of mice to act on GRIK5. Nucleosides Nucleotides Nucleic Acids 41, 463–473. doi: 10.1080/15257770.2022.2051048, PMID: 35357273

[ref101] Marcello L. Cavaliere C. Colangelo A. M. Bianco M. R. Cirillo G. Alberghina L.. (2013). Remodelling of supraspinal neuroglial network in neuropathic pain is featured by a reactive gliosis of the nociceptive amygdala. Eur. J. Pain 17, 799–810. doi: 10.1002/j.1532-2149.2012.00255.x, PMID: 23193101

[ref102] Martins I. Carvalho P. de Vries M. G. Teixeira-Pinto A. Wilson S. P. Westerink B. H. C.. (2015). GABA acting on GABAB receptors located in a medullary pain facilitatory area enhances nociceptive behaviors evoked by intraplantar formalin injection. Pain 156, 1555–1565. doi: 10.1097/j.pain.0000000000000203, PMID: 25932688

[ref103] Masocha W. (2015). Comprehensive analysis of the GABAergic system gene expression profile in the anterior cingulate cortex of mice with paclitaxel-induced neuropathic pain. Gene Expr. 16, 145–153. doi: 10.3727/105221615X14181438356337, PMID: 25700370 PMC8750099

[ref104] Mawla I. Ichesco E. Zöllner H. J. Edden R. A. E. Chenevert T. Buchtel H.. (2021). Greater somatosensory Afference with acupuncture increases primary somatosensory connectivity and alleviates fibromyalgia pain via insular γ-aminobutyric acid: a randomized neuroimaging trial. Arthritis Rheumatol. 73, 1318–1328. doi: 10.1002/art.41620, PMID: 33314799 PMC8197768

[ref105] Mazo C. Nissant A. Saha S. Peroni E. Lledo P. M. Lepousez G. (2022). Long-range GABAergic projections contribute to cortical feedback control of sensory processing. Nat. Commun. 13:6879. doi: 10.1038/s41467-022-34513-0, PMID: 36371430 PMC9653434

[ref106] Mecca C. M. Chao D. Yu G. Feng Y. Segel I. Zhang Z.. (2021). Dynamic change of endocannabinoid signaling in the medial prefrontal cortex controls the development of depression after neuropathic pain. J. Neurosci. 41, 7492–7508. doi: 10.1523/JNEUROSCI.3135-20.2021, PMID: 34244365 PMC8412994

[ref107] Meng X. Yue L. Liu A. Tao W. Shi L. Zhao W.. (2022). Distinct basolateral amygdala excitatory inputs mediate the somatosensory and aversive-affective components of pain. J. Biol. Chem. 298:102207. doi: 10.1016/j.jbc.2022.102207, PMID: 35772494 PMC9304789

[ref108] Mercer Lindsay N. Chen C. Gilam G. Mackey S. Scherrer G. (2021). Brain circuits for pain and its treatment. Sci. Transl. Med. 13:7360. doi: 10.1126/scitranslmed.abj7360, PMID: 34757810 PMC8675872

[ref109] Minami K. Kami K. Nishimura Y. Kawanishi M. Imashiro K. Kami T.. (2023). Voluntary running-induced activation of ventral hippocampal GABAergic interneurons contributes to exercise-induced hypoalgesia in neuropathic pain model mice. Sci. Rep. 13:2645. doi: 10.1038/s41598-023-29849-6, PMID: 36788313 PMC9929335

[ref110] Namgung J. Y. Noh E. Jang Y. Lee M. J. Park B. Y. (2025). A robust multimodal brain MRI-based diagnostic model for migraine: validation across different migraine phases and longitudinal follow-up data. J. Headache Pain 26:5. doi: 10.1186/s10194-024-01946-5, PMID: 39789428 PMC11716046

[ref111] Nashawi H. Masocha W. Edafiogho I. O. Kombian S. B. (2016). Paclitaxel causes electrophysiological changes in the anterior cingulate cortex via modulation of the γ-aminobutyric acid-ergic system. Med. Princ. Pract. 25, 423–428. doi: 10.1159/000447775, PMID: 27336416 PMC5588502

[ref112] Nicholls D. Attwell D. (1990). The release and uptake of excitatory amino acids. Trends Pharmacol. Sci. 11, 462–468. doi: 10.1016/0165-6147(90)90129-V, PMID: 1980041

[ref113] Niddam D. M. Wang S. J. Tsai S. Y. (2021). Pain sensitivity and the primary sensorimotor cortices: a multimodal neuroimaging study. Pain 162, 846–855. doi: 10.1097/j.pain.0000000000002074, PMID: 32947544

[ref114] Notartomaso S. Antenucci N. Mazzitelli M. Rovira X. Boccella S. Ricciardi F.. (2024). A 'double-edged' role for type-5 metabotropic glutamate receptors in pain disclosed by light-sensitive drugs. eLife 13:94931. doi: 10.7554/eLife.94931, PMID: 39172042 PMC11341090

[ref115] Obermann M. Rodriguez-Raecke R. Naegel S. Holle D. Mueller D. Yoon M. S.. (2013). Gray matter volume reduction reflects chronic pain in trigeminal neuralgia. NeuroImage 74, 352–358. doi: 10.1016/j.neuroimage.2013.02.029, PMID: 23485849

[ref116] Onderwater G. L. J. Wijnen J. P. Najac C. van Dongen R. M. Ronen I. Webb A.. (2021). Cortical glutamate and gamma-aminobutyric acid over the course of a provoked migraine attack, a 7 tesla magnetic resonance spectroscopy study. Neuroimage Clin. 32:102889. doi: 10.1016/j.nicl.2021.102889, PMID: 34911195 PMC8640106

[ref117] Ong W. Y. Stohler C. S. Herr D. R. (2019). Role of the prefrontal cortex in pain processing. Mol. Neurobiol. 56, 1137–1166. doi: 10.1007/s12035-018-1130-9, PMID: 29876878 PMC6400876

[ref118] Ozawa S. Kamiya H. Tsuzuki K. (1998). Glutamate receptors in the mammalian central nervous system. Prog. Neurobiol. 54, 581–618. doi: 10.1016/S0301-0082(97)00085-3, PMID: 9550192

[ref119] Palmer A. M. Robichaud P. J. Reiter C. T. (1994). The release and uptake of excitatory amino acids in rat brain: effect of aging and oxidative stress. Neurobiol. Aging 15, 103–111. doi: 10.1016/0197-4580(94)90150-3, PMID: 7909140

[ref120] Park S. H. Baker A. K. Krishna V. Mackey S. C. Martucci K. T. (2022). Altered resting-state functional connectivity within corticostriatal and subcortical-striatal circuits in chronic pain. Sci. Rep. 12:12683. doi: 10.1038/s41598-022-16835-7, PMID: 35879602 PMC9314446

[ref121] Peek A. L. Leaver A. M. Foster S. Puts N. A. Oeltzschner G. Henderson L.. (2021). Increase in ACC GABA+ levels correlate with decrease in migraine frequency, intensity and disability over time. J. Headache Pain 22:150. doi: 10.1186/s10194-021-01352-1, PMID: 34903165 PMC8903525

[ref122] Peek A. L. Rebbeck T. Puts N. A. Watson J. Aguila M. R. Leaver A. M. (2020). Brain GABA and glutamate levels across pain conditions: a systematic literature review and meta-analysis of 1H-MRS studies using the MRS-Q quality assessment tool. NeuroImage 210:116532. doi: 10.1016/j.neuroimage.2020.116532, PMID: 31958584

[ref123] Perea G. Navarrete M. Araque A. (2009). Tripartite synapses: astrocytes process and control synaptic information. Trends Neurosci. 32, 421–431. doi: 10.1016/j.tins.2009.05.001, PMID: 19615761

[ref124] Petroff O. A. (2002). GABA and glutamate in the human brain. Neuroscientist 8, 562–573. doi: 10.1177/1073858402238515, PMID: 12467378

[ref125] Pigott T. McPeak A. de Chastelain A. DeMayo M. M. Rasic N. Rayner L.. (2023). Changes in brain GABA and glutamate and improvements in physical functioning following intensive pain rehabilitation in youth with chronic pain. J. Pain 24, 1288–1297. doi: 10.1016/j.jpain.2023.02.027, PMID: 36966034

[ref126] Piot L. Heroven C. Bossi S. Zamith J. Malinauskas T. Johnson C.. (2023). GluD1 binds GABA and controls inhibitory plasticity. Science 382, 1389–1394. doi: 10.1126/science.adf3406, PMID: 38060673

[ref127] Qian X. Zhao X. Yu L. Yin Y. Zhang X. D. Wang L.. (2023). Current status of GABA receptor subtypes in analgesia. Biomed. Pharmacother. 168:115800. doi: 10.1016/j.biopha.2023.115800, PMID: 37935070

[ref128] Quidé Y. Jahanshad N. Andoh J. Antoniou G. Apkarian A. V. Ashar Y. K.. (2024). ENIGMA-chronic pain: a worldwide initiative to identify brain correlates of chronic pain. Pain 165, 2662–2666. doi: 10.1097/j.pain.0000000000003317, PMID: 39058957 PMC11562752

[ref129] Raja S. N. Carr D. B. Cohen M. Finnerup N. B. Flor H. Gibson S.. (2020). The revised International Association for the Study of Pain definition of pain: concepts, challenges, and compromises. Pain 161, 1976–1982. doi: 10.1097/j.pain.0000000000001939, PMID: 32694387 PMC7680716

[ref130] Rankin G. Chirila A. M. Emanuel A. J. Zhang Z. Woolf C. J. Drugowitsch J.. (2024). Nerve injury disrupts temporal processing in the spinal cord dorsal horn through alterations in PV(+) interneurons. Cell Rep. 43:113718. doi: 10.1016/j.celrep.2024.113718, PMID: 38294904 PMC11101906

[ref131] Reid P. Scherer K. Halasz D. Simal A. L. Tang J. Zaheer F.. (2025). Astrocyte neuronal metabolic coupling in the anterior cingulate cortex of mice with inflammatory pain. Brain Behav. Immun. 125, 212–225. doi: 10.1016/j.bbi.2024.12.025, PMID: 39694343

[ref132] Ren D. Li J. N. Qiu X. T. Wan F. P. Wu Z. Y. Fan B. Y.. (2022). Anterior cingulate cortex mediates hyperalgesia and anxiety induced by chronic pancreatitis in rats. Neurosci. Bull. 38, 342–358. doi: 10.1007/s12264-021-00800-x, PMID: 34907496 PMC9068840

[ref133] Romanos J. Benke D. Pietrobon D. Zeilhofer H. U. Santello M. (2020). Astrocyte dysfunction increases cortical dendritic excitability and promotes cranial pain in familial migraine. Sci. Adv. 6:1584. doi: 10.1126/sciadv.aaz1584, PMID: 32548257 PMC7274778

[ref134] Saffarpour S. Shaabani M. Naghdi N. Farahmandfar M. Janzadeh A. Nasirinezhad F. (2017). In vivo evaluation of the hippocampal glutamate, GABA and the BDNF levels associated with spatial memory performance in a rodent model of neuropathic pain. Physiol. Behav. 175, 97–103. doi: 10.1016/j.physbeh.2017.03.025, PMID: 28336100

[ref135] Sanganahalli B. G. Chitturi J. Herman P. Elkabes S. Heary R. Hyder F.. (2021). Supraspinal sensorimotor and pain-related reorganization after a Hemicontusion rat cervical spinal cord injury. J. Neurotrauma 38, 3393–3405. doi: 10.1089/neu.2021.0190, PMID: 34714150 PMC8713267

[ref136] Shackman A. J. Salomons T. V. Slagter H. A. Fox A. S. Winter J. J. Davidson R. J. (2011). The integration of negative affect, pain and cognitive control in the cingulate cortex. Nat. Rev. Neurosci. 12, 154–167. doi: 10.1038/nrn2994, PMID: 21331082 PMC3044650

[ref137] Shao F. B. Fang J. F. Wang S. S. Qiu M. T. Xi D. N. Jin X. M.. (2021). Anxiolytic effect of GABAergic neurons in the anterior cingulate cortex in a rat model of chronic inflammatory pain. Mol. Brain 14:139. doi: 10.1186/s13041-021-00849-9, PMID: 34507588 PMC8431944

[ref138] Shao S. Zheng Y. Fu Z. Wang J. Zhang Y. Wang C.. (2023). Ventral hippocampal CA1 modulates pain behaviors in mice with peripheral inflammation. Cell Rep. 42:112017. doi: 10.1016/j.celrep.2023.112017, PMID: 36662622

[ref139] Shen W. Chen F. Tang Y. Zhao Y. Zhu L. Xiang L.. (2025). mGluR5-mediated astrocytes hyperactivity in the anterior cingulate cortex contributes to neuropathic pain in male mice. Commun Biol. 8:266. doi: 10.1038/s42003-025-07733-5, PMID: 39979531 PMC11842833

[ref140] Singh A. Patel D. Li A. Hu L. Zhang Q. Liu Y.. (2020). Mapping cortical integration of sensory and affective pain pathways. Curr. Biol. 30, 1703–1715. doi: 10.1016/j.cub.2020.02.091, PMID: 32220320 PMC7224326

[ref141] Smith M. L. Asada N. Malenka R. C. (2021). Anterior cingulate inputs to nucleus accumbens control the social transfer of pain and analgesia. Science 371, 153–159. doi: 10.1126/science.abe3040, PMID: 33414216 PMC7952019

[ref142] Song Q. Wei A. Xu H. Gu Y. Jiang Y. Dong N.. (2024). An ACC-VTA-ACC positive-feedback loop mediates the persistence of neuropathic pain and emotional consequences. Nat. Neurosci. 27, 272–285. doi: 10.1038/s41593-023-01519-w, PMID: 38172439

[ref143] Stærmose T. G. Knudsen M. K. Kasch H. Blicher J. U. (2019). Cortical GABA in migraine with aura -an ultrashort echo magnetic resonance spectroscopy study. J. Headache Pain 20:110. doi: 10.1186/s10194-019-1059-z, PMID: 31795972 PMC6889606

[ref144] Stegemann A. Liu S. Retana Romero O. A. Oswald M. J. Han Y. Beretta C. A.. (2023). Prefrontal engrams of long-term fear memory perpetuate pain perception. Nat. Neurosci. 26, 820–829. doi: 10.1038/s41593-023-01291-x, PMID: 37024573 PMC10166861

[ref145] Sun T. Wang J. Li X. Li Y. J. Feng D. Shi W. L.. (2016). Gastrodin relieved complete Freund's adjuvant-induced spontaneous pain by inhibiting inflammatory response. Int. Immunopharmacol. 41, 66–73. doi: 10.1016/j.intimp.2016.10.020, PMID: 27816787

[ref146] Takeda I. Yoshihara K. Cheung D. L. Kobayashi T. Agetsuma M. Tsuda M.. (2022). Controlled activation of cortical astrocytes modulates neuropathic pain-like behaviour. Nat. Commun. 13:4100. doi: 10.1038/s41467-022-31773-8, PMID: 35835747 PMC9283422

[ref147] Talbot J. D. Marrett S. Evans A. C. Meyer E. Bushnell M. C. Duncan G. H. (1991). Multiple representations of pain in human cerebral cortex. Science 251, 1355–1358. doi: 10.1126/science.2003220, PMID: 2003220

[ref148] Tan L. L. Kuner R. (2021). Neocortical circuits in pain and pain relief. Nat. Rev. Neurosci. 22, 458–471. doi: 10.1038/s41583-021-00468-2, PMID: 34127843

[ref149] Tan L. Tring E. Ringach D. L. Zipursky S. L. Trachtenberg J. T. (2020). Vision changes the cellular composition of binocular circuitry during the critical period. Neuron 108, 735–47.e6. doi: 10.1016/j.neuron.2020.09.022, PMID: 33091339 PMC7704707

[ref150] Temmermand R. Barrett J. E. Fontana A. C. K. (2022). Glutamatergic systems in neuropathic pain and emerging non-opioid therapies. Pharmacol. Res. 185:106492. doi: 10.1016/j.phrs.2022.106492, PMID: 36228868 PMC10413816

[ref151] Thiaucourt M. Shabes P. Schloss N. Sack M. Baumgärtner U. Schmahl C.. (2018). Posterior insular GABA levels inversely correlate with the intensity of experimental mechanical pain in healthy subjects. Neuroscience 387, 116–122. doi: 10.1016/j.neuroscience.2017.09.043, PMID: 28978415

[ref152] Thompson J. M. Neugebauer V. (2019). Cortico-limbic pain mechanisms. Neurosci. Lett. 702, 15–23. doi: 10.1016/j.neulet.2018.11.037, PMID: 30503916 PMC6520155

[ref153] Tremblay R. Lee S. Rudy B. (2016). GABAergic interneurons in the neocortex: from cellular properties to circuits. Neuron 91, 260–292. doi: 10.1016/j.neuron.2016.06.033, PMID: 27477017 PMC4980915

[ref154] Tullis J. E. Larsen M. E. Rumian N. L. Freund R. K. Boxer E. E. Brown C. N.. (2023). LTP induction by structural rather than enzymatic functions of CaMKII. Nature 621, 146–153. doi: 10.1038/s41586-023-06465-y, PMID: 37648853 PMC10482691

[ref155] Vélez-Fort M. Audinat E. Angulo M. C. (2012). Central role of GABA in neuron-glia interactions. Neuroscientist 18, 237–250. doi: 10.1177/1073858411403317, PMID: 21609943

[ref156] Wang L. Pu H. Zhou J. Liu W. Zhang S. Tan Q.. (2024). Abnormal metabolites in the dorsolateral prefrontal cortex of female epilepsy patients with migraine without aura. Neuroreport 35, 1155–1162. doi: 10.1097/WNR.0000000000002110, PMID: 39526657 PMC11540266

[ref157] Wang J. Tu J. Cao B. Mu L. Yang X. Cong M.. (2017). Astrocytic l-lactate signaling facilitates amygdala-anterior cingulate cortex synchrony and decision making in rats. Cell Rep. 21, 2407–2418. doi: 10.1016/j.celrep.2017.11.012, PMID: 29186680

[ref158] Wang W. Zhang X. Bai X. Zhang Y. Yuan Z. Tang H.. (2022). Gamma-aminobutyric acid and glutamate/glutamine levels in the dentate nucleus and periaqueductal gray with episodic and chronic migraine: a proton magnetic resonance spectroscopy study. J. Headache Pain 23:83. doi: 10.1186/s10194-022-01452-6, PMID: 35840907 PMC9287958

[ref159] Waqas M. Gao S. Iram Us S. Ali M. K. Ma Y. Li W. (2018). Inner ear hair cell protection in mammals against the noise-induced cochlear damage. Neural Plast. 2018:3170801. doi: 10.1155/2018/3170801 30123244 PMC6079343

[ref160] Watson C. J. (2016). Insular balance of glutamatergic and GABAergic signaling modulates pain processing. Pain 157, 2194–2207. doi: 10.1097/j.pain.0000000000000615, PMID: 27218870

[ref161] Wei X. Centeno M. V. Ren W. Borruto A. M. Procissi D. Xu T.. (2021). Activation of the dorsal, but not the ventral, hippocampus relieves neuropathic pain in rodents. Pain 162, 2865–2880. doi: 10.1097/j.pain.0000000000002279, PMID: 34160168 PMC8464622

[ref162] Wei N. Guo Z. Qiu M. Ye R. Shao X. Liang Y.. (2024). Astrocyte activation in the ACC contributes to comorbid anxiety in chronic inflammatory pain and involves in the excitation-inhibition imbalance. Mol. Neurobiol. 61, 6934–6949. doi: 10.1007/s12035-024-04027-5, PMID: 38363535

[ref163] Wei X. Y. Wang X. Shi G. X. Tu J. F. Yang J. W. Ren M. M.. (2024). Acupuncture modulation of chronic neuropathic pain and its association with brain functional properties. J. Pain 25:104645. doi: 10.1016/j.jpain.2024.104645, PMID: 39089662

[ref164] Weizman L. Dayan L. Brill S. Nahman-Averbuch H. Hendler T. Jacob G.. (2018). Cannabis analgesia in chronic neuropathic pain is associated with altered brain connectivity. Neurology 91, e1285–e1294. doi: 10.1212/WNL.0000000000006293, PMID: 30185448 PMC6177269

[ref165] Wen Y. Dong Z. Liu J. Axerio-Cilies P. Du Y. Li J.. (2022). Glutamate and GABA(a) receptor crosstalk mediates homeostatic regulation of neuronal excitation in the mammalian brain. Signal Transduct. Target. Ther. 7:340. doi: 10.1038/s41392-022-01148-y, PMID: 36184627 PMC9527238

[ref166] Widman A. J. Bashar T. Burton A. Clausen D. M. Gupta P. Wolf D. K.. (2025). Chronic, battery-free, fully implantable multimodal spinal cord stimulator for pain modulation in small animal models. Adv. Sci. 12:e2415963. doi: 10.1002/advs.202415963, PMID: 40184607 PMC12140358

[ref167] Wu Y. Fu D. Gu Q. Li Y. Qian Z. Han J.. (2020). Activation of CB1 receptors on GABAergic interneurons in the ventrolateral orbital cortex induces analgesia. Neurosci. Lett. 736:135286. doi: 10.1016/j.neulet.2020.135286, PMID: 32745558

[ref168] Wu S. Ren X. Zhu C. Wang W. Zhang K. Li Z.. (2022). A c-Fos activation map in nitroglycerin/levcromakalim-induced models of migraine. J. Headache Pain 23:128. doi: 10.1186/s10194-022-01496-8, PMID: 36180824 PMC9524028

[ref169] Wu L. J. Steenland H. W. Kim S. S. Isiegas C. Abel T. Kaang B. K.. (2008). Enhancement of presynaptic glutamate release and persistent inflammatory pain by increasing neuronal cAMP in the anterior cingulate cortex. Mol. Pain 4:40. doi: 10.1186/1744-8069-4-40, PMID: 18823548 PMC2570662

[ref170] Wu X. Q. Tan B. Du Y. Yang L. Hu T. T. Ding Y. L.. (2023). Glutamatergic and GABAergic neurons in the vLGN mediate the nociceptive effects of green and red light on neuropathic pain. Neurobiol. Dis. 183:106164. doi: 10.1016/j.nbd.2023.106164, PMID: 37217103

[ref171] Xiao S. Sun H. Zhu Y. Shen Z. Zhu X. Yao P. A.. (2023). Electroacupuncture alleviates the relapse of pain-related aversive memory by activating KOR and inhibiting GABAergic neurons in the insular cortex. Cereb. Cortex 33, 10711–10721. doi: 10.1093/cercor/bhad321, PMID: 37679857 PMC10560575

[ref172] Yang S. Chang M. C. (2019). Chronic pain: structural and functional changes in brain structures and associated negative affective states. Int. J. Mol. Sci. 20:130. doi: 10.3390/ijms20133130, PMID: 31248061 PMC6650904

[ref173] Yin J. B. Liang S. H. Li F. Zhao W. J. Bai Y. Sun Y.. (2020). dmPFC-vlPAG projection neurons contribute to pain threshold maintenance and antianxiety behaviors. J. Clin. Invest. 130, 6555–6570. doi: 10.1172/JCI127607, PMID: 32841213 PMC7685740

[ref174] Zanetti L. Regoni M. Ratti E. Valtorta F. Sassone J. (2021). Presynaptic AMPA receptors in health and disease. Cells 10:2260. doi: 10.3390/cells10092260, PMID: 34571906 PMC8470629

[ref175] Zeng F. Zhang Q. Liu Y. Sun G. Li A. Talay R. S.. (2021). AMPAkines potentiate the corticostriatal pathway to reduce acute and chronic pain. Mol. Brain 14:45. doi: 10.1186/s13041-021-00757-y, PMID: 33653395 PMC7923831

[ref176] Zhang Z. Gadotti V. M. Chen L. Souza I. A. Stemkowski P. L. Zamponi G. W. (2015). Role of Prelimbic GABAergic circuits in sensory and emotional aspects of neuropathic pain. Cell Rep. 12, 752–759. doi: 10.1016/j.celrep.2015.07.001, PMID: 26212331

[ref177] Zhang M. M. Geng A. Q. Chen K. Wang J. Wang P. Qiu X. T.. (2022). Glutamatergic synapses from the insular cortex to the basolateral amygdala encode observational pain. Neuron 110, 1993–2008. doi: 10.1016/j.neuron.2022.03.030, PMID: 35443154

[ref178] Zhang Y. Li X. Qiu S. Jin R. Peng W. (2025). Preemptive transcranial direct current stimulation mitigates susceptibility to persistent pain. Commun. Biol. 8:865. doi: 10.1038/s42003-025-08304-4, PMID: 40473976 PMC12141659

[ref179] Zhang M. Li C. Xue Q. Lu C. B. Zhao H. Meng F. C.. (2023). Activation of cannabinoid receptor 1 in GABAergic neurons in the rostral anterior insular cortex contributes to the analgesia following common peroneal nerve ligation. Neurosci. Bull. 39, 1348–1362. doi: 10.1007/s12264-023-01029-6, PMID: 36773215 PMC10465468

[ref180] Zhang Q. Manders T. Tong A. P. Yang R. Garg A. Martinez E.. (2017). Chronic pain induces generalized enhancement of aversion. eLife 6:25302. doi: 10.7554/eLife.25302, PMID: 28524819 PMC5438248

[ref181] Zhang H. Zhao L. Lu X. Peng W. Zhang L. Zhang Z.. (2024). Multimodal covarying brain patterns mediate genetic and psychological contributions to individual differences in pain sensitivity. Pain 165, 1074–1085. doi: 10.1097/j.pain.0000000000003103, PMID: 37943083

[ref182] Zheng Y. Zhou Y. Wu Q. Yue J. Ying X. Li S.. (2020). Effect of electroacupuncture on the expression of P2 × 4, GABAA γ 2 and long-term potentiation in spinal cord of rats with neuropathic pain. Brain Res. Bull. 162, 1–10. doi: 10.1016/j.brainresbull.2020.04.020, PMID: 32428626

[ref183] Zhou W. Ye C. Wang H. Mao Y. Zhang W. Liu A.. (2022). Sound induces analgesia through corticothalamic circuits. Science 377, 198–204. doi: 10.1126/science.abn4663, PMID: 35857536 PMC9636983

[ref184] Zhou Q. Zhong Q. Liu Z. Zhao Z. Wang J. Zhang Z. (2025). Modulating anxiety-like behaviors in neuropathic pain: role of anterior cingulate cortex astrocytes activation. CNS Neurosci. Ther. 31:e70227. doi: 10.1111/cns.70227, PMID: 39838823 PMC11751476

[ref185] Ziegler K. Folkard R. Gonzalez A. J. Burghardt J. Antharvedi-Goda S. Martin-Cortecero J.. (2023). Primary somatosensory cortex bidirectionally modulates sensory gain and nociceptive behavior in a layer-specific manner. Nat. Commun. 14:2999. doi: 10.1038/s41467-023-38798-7, PMID: 37225702 PMC10209111

[ref186] Zielman R. Wijnen J. P. Webb A. Onderwater G. L. J. Ronen I. Ferrari M. D.. (2017). Cortical glutamate in migraine. Brain 140, 1859–1871. doi: 10.1093/brain/awx130, PMID: 28633367

[ref187] Zugaib J. Coutinho M. R. Ferreira M. D. Menescal-de-Oliveira L. (2014). Glutamate/GABA balance in ACC modulates the nociceptive responses of vocalization: an expression of affective-motivational component of pain in guinea pigs. Physiol. Behav. 126, 8–14. doi: 10.1016/j.physbeh.2013.12.004, PMID: 24382484

[ref188] Zunhammer M. Schweizer L. M. Witte V. Harris R. E. Bingel U. Schmidt-Wilcke T. (2016). Combined glutamate and glutamine levels in pain-processing brain regions are associated with individual pain sensitivity. Pain 157, 2248–2256. doi: 10.1097/j.pain.0000000000000634, PMID: 27649042

